# Supplementary material for: A REP-FAMSEC Method as a Tool in Explaining Reaction Mechanisms: A Nucleophilic Substitution of 2-Phenylquinoxaline as a DFT Case Study
Source: Molecules. 2021 Mar 12;26(6):1570. doi: 10.3390/molecules26061570 (PMC7998666; doi:10.3390/molecules26061570)
Supplement: Supplementary file 1 [file molecules-26-01570-s001.pdf]

Understanding Reaction Mechanisms from Fundamental Properties of Molecular Systems: A DFT Study of The Preferred Electrophilic Site for Nucleophilic Substitution of 2-Phenylquinoxaline

Brian Kamogelo Mdhluli,[1] Winston Nxumalo[2] and Ignacy Cukrowski[1*]

1) *Department of Chemistry, Faculty of Natural and Agricultural Sciences, University of Pretoria, Lynnwood Road, Hatfield, Pretoria 0002, South Africa*
2) *Department of Chemistry, Faculty of Science and Agriculture, University of Limpopo, Private Bag X1106, Sovenga 0727, South Africa*

*Correspondence to: Ignacy Cukrowski

E-mail: ignacy.cukrowski@up.ac.za

SUPPLEMENTARY INFORMATION

Table of content

Part 1<br>XYZ coordinates of molecular systems (Tables S1-S22) and their energies (Tables S23-S31)	S2
Part 2<br>Selected geometric data	S27
Part 3<br>Most attractive and repulsive diatomic interactions in **3**, **4** and **5** along four reaction pathways	S36
Part 4<br>Net atomic charges	S39
Part 5<br>Inter-fragment interaction energies	S47
Part 6<br>Energy profiles along reaction coordinates	S61

Part 1

XYZ coordinates of molecular systems (Tables S1-S22) and their energies (Tables S23-S31)

Table S1. Cartesian coordinates for 2-phenylquinoxaline (**1**). The structure was optimized at the RB3LYP-GD3/6-311++G(d,p)/THF level of theory at 195.15 K. Coordinate values are given in Å.

Centre number	Atomic number	Atom type	Coordinates		
			X	Y	Z
1	7	N	-2.561578	-5.391291	1.045448
2	6	C	-3.171860	-6.543670	0.840572
3	6	C	-2.414088	-7.727561	0.558092
4	7	N	-1.105299	-7.744966	0.509644
5	6	C	-0.459390	-6.564839	0.738712
6	6	C	0.954497	-6.514524	0.707871
7	6	C	1.603252	-5.321337	0.928172
8	6	C	0.867555	-4.137588	1.184443
9	6	C	-0.507244	-4.158598	1.220148
10	6	C	-1.202551	-5.373876	0.998761
11	1	H	-2.925606	-8.661385	0.348908
12	1	H	1.498488	-7.430306	0.508183
13	1	H	2.686007	-5.280968	0.905732
14	1	H	1.398612	-3.208124	1.354225
15	1	H	-1.085534	-3.263325	1.416276
16	6	C	-4.653299	-6.577944	0.880014
17	6	C	-5.387646	-5.432690	0.535766
18	6	C	-6.778290	-5.448719	0.564783
19	6	C	-7.460046	-6.606220	0.945821
20	6	C	-6.739983	-7.747173	1.298108
21	6	C	-5.347164	-7.735668	1.262930
22	1	H	-4.856216	-4.536676	0.240437
23	1	H	-7.332119	-4.558830	0.287779
24	1	H	-8.543756	-6.617430	0.969693
25	1	H	-7.261274	-8.646153	1.606462
26	1	H	-4.805263	-8.625147	1.561374

Table S2. Cartesian coordinates for lithium phenylacetylide (**2**). The structure was optimized at the RB3LYP-GD3/6-311++G(d,p)/THF level of theory at 195.15 K. Coordinate values are given in Å.

Centre number	Atomic number	Atom type	Coordinates		
			X	Y	Z
1	3	Li	-5.551269	6.899123	1.149833
2	6	C	-6.409846	5.503681	-0.058057
3	6	C	-6.975264	4.668254	-0.769355
4	6	C	-7.640430	3.701114	-1.585653
5	6	C	-8.981622	3.884529	-1.981672
6	6	C	-9.630771	2.939738	-2.771411
7	6	C	-8.962615	1.786883	-3.188788
8	6	C	-7.633981	1.590470	-2.807056
9	6	C	-6.980019	2.532275	-2.017420
10	1	H	-9.506335	4.776963	-1.660455
11	1	H	-10.662928	3.103583	-3.062016
12	1	H	-9.470416	1.051774	-3.802741
13	1	H	-7.104576	0.698839	-3.125411
14	1	H	-5.948772	2.372884	-1.724138

Table S3. Cartesian coordinates for the adduct along RP-C2 (**3a**). The structure was optimized at the RB3LYP-GD3/6-311++G(d,p)/THF level of theory at 195.15 K. Coordinate values are given in Å.

Centre number	Atomic number	Atom type	Coordinates		
			X	Y	Z
1	7	N	-2.677838	-4.810929	-0.347156
2	6	C	-3.868354	-5.315044	-0.060273
3	6	C	-4.163881	-5.772890	1.263509
4	7	N	-3.302638	-5.726831	2.247123
5	6	C	-2.062474	-5.234618	1.964185
6	6	C	-1.084480	-5.173158	2.984877
7	6	C	0.173183	-4.692307	2.704648
8	6	C	0.499748	-4.261112	1.396425
9	6	C	-0.433836	-4.305201	0.386766
10	6	C	-1.740522	-4.786391	0.647929
11	1	H	-5.142451	-6.189645	1.476597
12	1	H	-1.356224	-5.515702	3.976246
13	1	H	0.923371	-4.644935	3.485020
14	1	H	1.498444	-3.894466	1.190200
15	1	H	-0.179657	-3.985844	-0.617376
16	6	C	-4.889064	-5.425077	-1.123854
17	6	C	-4.509917	-5.777076	-2.427651
18	6	C	-5.464234	-5.881841	-3.435373
19	6	C	-6.808573	-5.633913	-3.153697
20	6	C	-7.193576	-5.286944	-1.858364
21	6	C	-6.243216	-5.189700	-0.846490
22	1	H	-3.466861	-5.981625	-2.639707
23	1	H	-5.161144	-6.162639	-4.437570
24	1	H	-7.551566	-5.711747	-3.939206
25	1	H	-8.234064	-5.080181	-1.636459
26	1	H	-6.549952	-4.883538	0.145659
27	3	Li	-2.677762	-3.061115	-1.721302
28	6	C	-4.052338	-1.817809	-0.868748
29	6	C	-5.002988	-1.910774	-0.087462
30	6	C	-6.080786	-2.175201	0.809545
31	6	C	-5.837257	-2.785822	2.057632
32	6	C	-6.887854	-3.128875	2.903844
33	6	C	-8.208209	-2.865508	2.533526
34	6	C	-8.465350	-2.249936	1.306267
35	6	C	-7.418632	-1.908225	0.454905
36	1	H	-4.815298	-3.001095	2.345941
37	1	H	-6.675114	-3.606331	3.854334
38	1	H	-9.025784	-3.134830	3.192583
39	1	H	-9.487322	-2.039754	1.009348
40	1	H	-7.625066	-1.444327	-0.502541

Table S4. Cartesian coordinates for the transition state along RP-C2 (**4a**). The structure was optimized at the RB3LYP-GD3/6-311++G(d,p)/THF level of theory at 195.15 K. Coordinate values are given in Å.

Centre number	Atomic number	Atom type	Coordinates		
			X	Y	Z
1	7	N	-2.514329	-4.745019	-0.731607
2	6	C	-3.845762	-4.972955	-0.448143
3	6	C	-4.129752	-5.747665	0.791890
4	7	N	-3.283939	-5.905038	1.747543
5	6	C	-2.020273	-5.348714	1.581820
6	6	C	-1.098321	-5.401230	2.638844
7	6	C	0.192609	-4.923554	2.475349
8	6	C	0.583361	-4.407145	1.225813
9	6	C	-0.307893	-4.355884	0.168060
10	6	C	-1.643604	-4.806219	0.318389
11	1	H	-5.118137	-6.187819	0.898672
12	1	H	-1.424832	-5.828417	3.581163
13	1	H	0.898833	-4.958513	3.296482
14	1	H	1.597093	-4.045992	1.088062
15	1	H	-0.001033	-3.966712	-0.797532
16	6	C	-4.685295	-5.400694	-1.642921
17	6	C	-4.049101	-5.851069	-2.803724
18	6	C	-4.794102	-6.306672	-3.892959
19	6	C	-6.185512	-6.333850	-3.829425
20	6	C	-6.828216	-5.897289	-2.668677
21	6	C	-6.085254	-5.430250	-1.588316
22	1	H	-2.967450	-5.848326	-2.838783
23	1	H	-4.283191	-6.648529	-4.786436
24	1	H	-6.765354	-6.692499	-4.672490
25	1	H	-7.910766	-5.913868	-2.608097
26	1	H	-6.595048	-5.066988	-0.704749
27	3	Li	-2.966423	-2.860961	-1.278682
28	6	C	-4.666444	-3.337147	0.032980
29	6	C	-5.535462	-2.914449	0.794217
30	6	C	-6.544643	-2.413824	1.662227
31	6	C	-6.249090	-2.099927	3.003952
32	6	C	-7.242848	-1.619454	3.850425
33	6	C	-8.545708	-1.444795	3.379210
34	6	C	-8.851500	-1.753918	2.051951
35	6	C	-7.863514	-2.232960	1.198462
36	1	H	-5.238366	-2.239914	3.368342
37	1	H	-7.002158	-1.382237	4.880526
38	1	H	-9.317779	-1.070591	4.041695
39	1	H	-9.861980	-1.620200	1.682543
40	1	H	-8.098357	-2.471662	0.167810

Table S5. Cartesian coordinates for the intermediate along RP-C2 (**5a**). The structure was optimized at the RB3LYP-GD3/6-311++G(d,p)/THF level of theory at 195.15 K. Coordinate values are given in Å.

Centre number	Atomic number	Atom type	Coordinates		
			X	Y	Z
1	7	N	-2.929642	-4.462909	-0.645011
2	6	C	-4.193668	-5.178884	-0.374334
3	6	C	-3.922862	-6.446356	0.458001
4	7	N	-2.988871	-6.526749	1.326356
5	6	C	-2.101556	-5.449661	1.436080
6	6	C	-1.181971	-5.430504	2.490857
7	6	C	-0.169611	-4.479871	2.547114
8	6	C	-0.055415	-3.559650	1.490838
9	6	C	-0.951262	-3.565499	0.433311
10	6	C	-2.040625	-4.479866	0.381661
11	1	H	-4.573197	-7.305040	0.297706
12	1	H	-1.273708	-6.193625	3.257701
13	1	H	0.529576	-4.462219	3.374721
14	1	H	0.743016	-2.823947	1.503067
15	1	H	-0.845740	-2.833128	-0.362022
16	6	C	-4.837731	-5.581701	-1.710369
17	6	C	-4.147885	-6.479488	-2.537387
18	6	C	-4.674489	-6.865090	-3.767597
19	6	C	-5.902744	-6.355506	-4.195550
20	6	C	-6.596641	-5.464745	-3.379802
21	6	C	-6.068402	-5.083709	-2.143329
22	1	H	-3.191410	-6.872112	-2.211492
23	1	H	-4.129404	-7.565179	-4.391178
24	1	H	-6.314216	-6.654587	-5.153063
25	1	H	-7.552623	-5.065351	-3.700337
26	1	H	-6.618211	-4.396526	-1.511411
27	3	Li	-2.834004	-3.415920	-2.286917
28	6	C	-5.096647	-4.326501	0.429792
29	6	C	-5.755500	-3.603108	1.140722
30	6	C	-6.530642	-2.751637	1.982479
31	6	C	-6.034461	-2.344356	3.234686
32	6	C	-6.791501	-1.512289	4.054021
33	6	C	-8.050871	-1.072769	3.642002
34	6	C	-8.551690	-1.471916	2.401619
35	6	C	-7.801354	-2.304169	1.576462
36	1	H	-5.057002	-2.686396	3.553360
37	1	H	-6.397773	-1.206675	5.016808
38	1	H	-8.637813	-0.425076	4.283091
39	1	H	-9.529668	-1.134964	2.076831
40	1	H	-8.191322	-2.615273	0.614399

Table S6. Cartesian coordinates for the adduct along RP-C3 (**3b**). The structure was optimized at the RB3LYP-GD3/6-311++G(d,p)/THF level of theory at 195.15 K. Coordinate values are given in Å.

Centre number	Atomic number	Atom type	Coordinates		
			X	Y	Z
1	7	N	-2.540885	-5.207869	0.754700
2	6	C	-3.165730	-6.200555	0.149768
3	6	C	-2.424778	-7.286464	-0.414816
4	7	N	-1.116636	-7.377704	-0.338431
5	6	C	-0.451149	-6.366488	0.300255
6	6	C	0.956599	-6.398878	0.423224
7	6	C	1.613215	-5.368042	1.056366
8	6	C	0.891074	-4.271301	1.586559
9	6	C	-0.479274	-4.217618	1.482119
10	6	C	-1.184210	-5.265181	0.838398
11	1	H	-2.927391	-8.089329	-0.940120
12	1	H	1.500631	-7.241876	0.012264
13	1	H	2.692373	-5.391859	1.151238
14	1	H	1.427956	-3.469414	2.079607
15	1	H	-1.048986	-3.388413	1.884290
16	6	C	-4.643143	-6.177398	0.041378
17	6	C	-5.338910	-4.960839	0.117112
18	6	C	-6.725086	-4.933126	0.002635
19	6	C	-7.440762	-6.118450	-0.183514
20	6	C	-6.760054	-7.333518	-0.251858
21	6	C	-5.372339	-7.363204	-0.141566
22	1	H	-4.782367	-4.042582	0.256862
23	1	H	-7.249272	-3.985483	0.053930
24	1	H	-8.520825	-6.092903	-0.274238
25	1	H	-7.299320	-8.263549	-0.390971
26	1	H	-4.871832	-8.321677	-0.184618
27	3	Li	-0.506003	-9.230987	-1.241854
28	6	C	-2.194178	-10.359079	-1.612780
29	6	C	-3.402037	-10.615066	-1.589208
30	6	C	-4.816343	-10.810757	-1.515669
31	6	C	-5.639583	-10.626027	-2.645283
32	6	C	-7.021966	-10.760743	-2.552664
33	6	C	-7.621789	-11.083440	-1.333890
34	6	C	-6.820700	-11.274943	-0.206474
35	6	C	-5.437630	-11.143655	-0.293887
36	1	H	-5.181059	-10.367437	-3.592691
37	1	H	-7.634470	-10.609057	-3.434885
38	1	H	-8.698959	-11.183263	-1.263261
39	1	H	-7.275449	-11.523765	0.746296
40	1	H	-4.821589	-11.285952	0.586593

Table S7. Cartesian coordinates for the transition state along RP-C3 (**4b**). The structure was optimized at the RB3LYP-GD3/6-311++G(d,p)/THF level of theory at 195.15 K. Coordinate values are given in Å.

Centre number	Atomic number	Atom type	Coordinates		
			X	Y	Z
1	7	N	-2.633552	-5.874902	0.622870
2	6	C	-3.117965	-6.350976	-0.483601
3	6	C	-2.226372	-7.015667	-1.479529
4	7	N	-0.873161	-6.745181	-1.419623
5	6	C	-0.392486	-6.324034	-0.214875
6	6	C	1.003543	-6.232361	0.017020
7	6	C	1.493062	-5.805547	1.238281
8	6	C	0.617539	-5.435493	2.278437
9	6	C	-0.749800	-5.483387	2.066320
10	6	C	-1.271571	-5.921724	0.834993
11	1	H	-2.601541	-7.011904	-2.497755
12	1	H	1.679482	-6.505142	-0.787081
13	1	H	2.565163	-5.752775	1.395746
14	1	H	1.013509	-5.102469	3.230602
15	1	H	-1.449856	-5.180574	2.837740
16	6	C	-4.583280	-6.285554	-0.705043
17	6	C	-5.423352	-5.822894	0.323840
18	6	C	-6.802914	-5.792534	0.162457
19	6	C	-7.382638	-6.223408	-1.033489
20	6	C	-6.563920	-6.678264	-2.064504
21	6	C	-5.180245	-6.709799	-1.902825
22	1	H	-4.974230	-5.499011	1.253508
23	1	H	-7.430511	-5.438586	0.972981
24	1	H	-8.459634	-6.207044	-1.156904
25	1	H	-7.000810	-7.020288	-2.995910
26	1	H	-4.575843	-7.087958	-2.716072
27	3	Li	-0.501947	-8.717651	-1.655249
28	6	C	-2.607248	-8.812688	-1.201167
29	6	C	-3.675066	-9.398424	-1.014024
30	6	C	-4.924598	-10.019480	-0.745510
31	6	C	-5.586405	-10.783153	-1.726685
32	6	C	-6.825548	-11.354420	-1.457037
33	6	C	-7.429288	-11.171054	-0.211239
34	6	C	-6.784129	-10.412675	0.768046
35	6	C	-5.541953	-9.844571	0.509318
36	1	H	-5.120630	-10.917743	-2.695807
37	1	H	-7.324102	-11.941205	-2.220313
38	1	H	-8.396749	-11.614675	-0.005538
39	1	H	-7.251418	-10.263761	1.734915
40	1	H	-5.042393	-9.248914	1.263622

Table S8. Cartesian coordinates for the intermediate along RP-C3 (**5b**). The structure was optimized at the RB3LYP-GD3/6-311++G(d,p)/THF level of theory at 195.15 K. Coordinate values are given in Å.

Centre number	Atomic number	Atom type	Coordinates		
			X	Y	Z
1	7	N	-3.113721	-5.607284	0.503028
2	6	C	-3.656034	-5.886507	-0.635509
3	6	C	-3.084776	-7.025348	-1.493826
4	7	N	-1.619324	-7.045631	-1.424380
5	6	C	-1.137041	-6.866741	-0.170954
6	6	C	0.194301	-7.224322	0.180443
7	6	C	0.666995	-7.070639	1.472930
8	6	C	-0.144562	-6.520156	2.482989
9	6	C	-1.421313	-6.087209	2.152286
10	6	C	-1.925123	-6.236087	0.850824
11	1	H	-3.366406	-6.856858	-2.537767
12	1	H	0.830808	-7.666549	-0.581312
13	1	H	1.677461	-7.389134	1.710057
14	1	H	0.230985	-6.413777	3.493972
15	1	H	-2.054770	-5.603378	2.889263
16	6	C	-4.832345	-5.121846	-1.098951
17	6	C	-5.137404	-3.872547	-0.527447
18	6	C	-6.248907	-3.149537	-0.943132
19	6	C	-7.087415	-3.655281	-1.941053
20	6	C	-6.800835	-4.893116	-2.514135
21	6	C	-5.684590	-5.618761	-2.099786
22	1	H	-4.484254	-3.481310	0.242482
23	1	H	-6.462121	-2.185742	-0.493709
24	1	H	-7.953492	-3.089769	-2.266015
25	1	H	-7.449494	-5.299764	-3.282150
26	1	H	-5.497433	-6.588988	-2.543650
27	3	Li	-0.590042	-7.415409	-3.029295
28	6	C	-3.687994	-8.311739	-1.083077
29	6	C	-4.148815	-9.372697	-0.729011
30	6	C	-4.688389	-10.616944	-0.285247
31	6	C	-5.583785	-11.343862	-1.091174
32	6	C	-6.105704	-12.555840	-0.648462
33	6	C	-5.745027	-13.064073	0.600588
34	6	C	-4.857205	-12.350435	1.407637
35	6	C	-4.331824	-11.137389	0.972759
36	1	H	-5.865284	-10.949536	-2.060529
37	1	H	-6.795989	-13.104484	-1.279348
38	1	H	-6.153500	-14.008094	0.942675
39	1	H	-4.574223	-12.739032	2.379421
40	1	H	-3.644260	-10.582073	1.599483

Table S9. Cartesian coordinates for the adduct along RP-C5 (**3c**). The structure was optimized at the RB3LYP-GD3/6-311++G(d,p)/THF level of theory at 195.15 K. Coordinate values are given in Å.

Centre number	Atomic number	Atom type	Coordinates		
			X	Y	Z
1	7	N	-2.648498	-4.774117	1.104270
2	6	C	-3.408317	-5.476974	0.285174
3	6	C	-2.815890	-6.356432	-0.674545
4	7	N	-1.517613	-6.529335	-0.778724
5	6	C	-0.713656	-5.825683	0.077856
6	6	C	0.692347	-5.973001	0.025264
7	6	C	1.482646	-5.246959	0.886410
8	6	C	0.905662	-4.353514	1.822627
9	6	C	-0.458252	-4.194558	1.892650
10	6	C	-1.301247	-4.929240	1.021789
11	1	H	-3.443608	-6.897798	-1.373696
12	1	H	1.121574	-6.658849	-0.698402
13	1	H	2.559982	-5.357564	0.849562
14	1	H	1.550154	-3.792286	2.489104
15	1	H	-0.918907	-3.518225	2.602636
16	6	C	-4.878983	-5.312091	0.356923
17	6	C	-5.423306	-4.089891	0.779553
18	6	C	-6.801794	-3.916569	0.847270
19	6	C	-7.659480	-4.962792	0.501761
20	6	C	-7.128499	-6.184446	0.089435
21	6	C	-5.748355	-6.358425	0.014016
22	1	H	-4.755535	-3.280121	1.045417
23	1	H	-7.208754	-2.963942	1.166605
24	1	H	-8.733538	-4.827067	0.556064
25	1	H	-7.787648	-7.005437	-0.168015
26	1	H	-5.354847	-7.322414	-0.286109
27	3	Li	-0.752249	-7.806490	-2.278752
28	6	C	1.169406	-8.354643	-2.685531
29	6	C	2.371729	-8.613925	-2.788932
30	6	C	3.768430	-8.901551	-2.888167
31	6	C	4.334975	-10.000764	-2.209825
32	6	C	5.696990	-10.273973	-2.300920
33	6	C	6.531961	-9.460996	-3.070068
34	6	C	5.987206	-8.369096	-3.748856
35	6	C	4.625854	-8.091327	-3.661134
36	1	H	3.692571	-10.635684	-1.610471
37	1	H	6.108772	-11.125134	-1.769220
38	1	H	7.592377	-9.675419	-3.139569
39	1	H	6.626012	-7.730640	-4.349704
40	1	H	4.209515	-7.241801	-4.190264

Table S10. Cartesian coordinates for the transition state along RP-C5 (**4c**). The structure was optimized at the RB3LYP-GD3/6-311++G(d,p)/THF level of theory at 195.15 K. Coordinate values are given in Å.

Centre number	Atomic number	Atom type	Coordinates		
			X	Y	Z
1	7	N	-1.601441	-5.546360	0.275462
2	6	C	-2.608378	-6.441369	0.213193
3	6	C	-2.528313	-7.613235	1.005340
4	7	N	-1.427132	-8.038598	1.600904
5	6	C	-0.241933	-7.325463	1.251033
6	6	C	0.839862	-7.523615	2.236865
7	6	C	1.754826	-6.553679	2.477026
8	6	C	1.661251	-5.269271	1.840471
9	6	C	0.564820	-4.950802	1.088637
10	6	C	-0.474153	-5.901767	0.870643
11	1	H	-3.437368	-8.169166	1.223035
12	1	H	0.885644	-8.487969	2.730252
13	1	H	2.559920	-6.733027	3.181702
14	1	H	2.433907	-4.530736	2.020442
15	1	H	0.432121	-3.958597	0.671326
16	6	C	-3.835126	-6.081242	-0.523779
17	6	C	-4.114556	-4.740398	-0.844791
18	6	C	-5.270411	-4.396031	-1.538917
19	6	C	-6.180774	-5.379199	-1.933126
20	6	C	-5.913233	-6.714621	-1.629993
21	6	C	-4.753393	-7.061968	-0.941109
22	1	H	-3.414166	-3.974493	-0.536906
23	1	H	-5.465124	-3.354201	-1.770141
24	1	H	-7.080795	-5.109233	-2.473833
25	1	H	-6.602990	-7.491212	-1.942457
26	1	H	-4.551995	-8.109380	-0.746944
27	3	Li	-0.879754	-9.671580	0.428887
28	6	C	0.413228	-8.025687	-0.265255
29	6	C	1.326900	-7.757774	-1.046339
30	6	C	2.359709	-7.435007	-1.964862
31	6	C	2.110345	-6.559150	-3.041994
32	6	C	3.127258	-6.227582	-3.930570
33	6	C	4.407114	-6.763345	-3.769846
34	6	C	4.665559	-7.634831	-2.709281
35	6	C	3.656083	-7.968847	-1.813181
36	1	H	1.117208	-6.143691	-3.164778
37	1	H	2.922046	-5.550018	-4.751730
38	1	H	5.196948	-6.503704	-4.465359
39	1	H	5.657822	-8.052255	-2.579859
40	1	H	3.857649	-8.640069	-0.986682

Table S11. Cartesian coordinates for the intermediate along RP-C5 (**5c**). The structure was optimized at the RB3LYP-GD3/6-311++G(d,p)/THF level of theory at 195.15 K. Coordinate values are given in Å.

Centre number	Atomic number	Atom type	Coordinates		
			X	Y	Z
1	7	N	-1.611383	-5.396214	0.812000
2	6	C	-2.299527	-6.383496	0.175461
3	6	C	-2.070066	-7.711985	0.595607
4	7	N	-1.014609	-8.116476	1.284671
5	6	C	0.080303	-7.112720	1.323383
6	6	C	1.010165	-7.458140	2.470060
7	6	C	1.472819	-6.526728	3.328854
8	6	C	1.049616	-5.147016	3.262897
9	6	C	0.065235	-4.775826	2.394324
10	6	C	-0.538891	-5.710579	1.499271
11	1	H	-2.869524	-8.441097	0.470302
12	1	H	1.345892	-8.489071	2.524541
13	1	H	2.168182	-6.819218	4.109804
14	1	H	1.475687	-4.428375	3.953078
15	1	H	-0.325779	-3.763371	2.385325
16	6	C	-3.423552	-6.023424	-0.698717
17	6	C	-3.959128	-4.719573	-0.702702
18	6	C	-5.026724	-4.381777	-1.528641
19	6	C	-5.598965	-5.331301	-2.379431
20	6	C	-5.073216	-6.624381	-2.397523
21	6	C	-3.997361	-6.962374	-1.580112
22	1	H	-3.525874	-3.979061	-0.042144
23	1	H	-5.419219	-3.370244	-1.506308
24	1	H	-6.430522	-5.065763	-3.022408
25	1	H	-5.491605	-7.370691	-3.064973
26	1	H	-3.582432	-7.962243	-1.644454
27	3	Li	-1.012352	-9.907698	2.116123
28	6	C	0.837420	-7.148174	0.046557
29	6	C	1.489060	-7.192458	-0.971661
30	6	C	2.231327	-7.232882	-2.189738
31	6	C	1.580649	-7.061104	-3.425689
32	6	C	2.303857	-7.103077	-4.613968
33	6	C	3.683521	-7.315017	-4.592337
34	6	C	4.338447	-7.484338	-3.371234
35	6	C	3.622184	-7.444820	-2.178532
36	1	H	0.510348	-6.893036	-3.441241
37	1	H	1.789790	-6.968320	-5.558972
38	1	H	4.244168	-7.345884	-5.519570
39	1	H	5.410172	-7.646707	-3.347755
40	1	H	4.131074	-7.574517	-1.230664

Table S12. Cartesian coordinates for the adduct along RP-C10 (**3d**). The structure was optimized at the RB3LYP-GD3/6-311++G(d,p)/THF level of theory at 195.15 K. Coordinate values are given in Å.

Centre number	Atomic number	Atom type	Coordinates		
			X	Y	Z
1	7	N	-2.161317	-4.919892	0.839698
2	6	C	-3.151169	-5.493258	0.172471
3	6	C	-2.881034	-6.470782	-0.835898
4	7	N	-1.670140	-6.844902	-1.162939
5	6	C	-0.635658	-6.249393	-0.503896
6	6	C	0.696028	-6.604104	-0.825465
7	6	C	1.743726	-6.003258	-0.167820
8	6	C	1.500704	-5.028722	0.830637
9	6	C	0.217151	-4.664977	1.166872
10	6	C	-0.880044	-5.270186	0.506656
11	1	H	-3.708621	-6.920963	-1.374697
12	1	H	0.856676	-7.349544	-1.595074
13	1	H	2.764237	-6.272565	-0.413361
14	1	H	2.339019	-4.562247	1.334718
15	1	H	0.023839	-3.915902	1.928293
16	6	C	-4.547747	-5.091409	0.456609
17	6	C	-4.874537	-3.734573	0.594801
18	6	C	-6.186588	-3.349997	0.858122
19	6	C	-7.186690	-4.314729	0.984631
20	6	C	-6.871007	-5.666129	0.839880
21	6	C	-5.560803	-6.054077	0.570817
22	1	H	-4.103120	-2.982975	0.470464
23	1	H	-6.429178	-2.298195	0.955266
24	1	H	-8.207710	-4.014802	1.190292
25	1	H	-7.644209	-6.418894	0.940252
26	1	H	-5.321157	-7.106837	0.473417
27	3	Li	-2.468607	-3.879250	2.693844
28	6	C	-1.301048	-2.518215	3.662500
29	6	C	-0.520284	-1.728813	4.201566
30	6	C	0.394182	-0.818267	4.816284
31	6	C	0.588067	-0.811444	6.213181
32	6	C	1.482298	0.074558	6.807669
33	6	C	2.207234	0.977994	6.027984
34	6	C	2.026853	0.984951	4.643332
35	6	C	1.134054	0.100984	4.043741
36	1	H	0.028237	-1.510078	6.824276
37	1	H	1.613872	0.060256	7.884329
38	1	H	2.902953	1.667207	6.492991
39	1	H	2.584187	1.682698	4.027495
40	1	H	0.998086	0.111605	2.968379

Table S13. Cartesian coordinates for the transition state along RP-C10 (**4d**). The structure was optimized at the RB3LYP-GD3/6-311++G(d,p)/THF level of theory at 195.15 K. Coordinate values are given in Å.

Centre number	Atomic number	Atom type	Coordinates		
			X	Y	Z
1	7	N	-2.729809	-3.451478	0.573532
2	6	C	-3.116273	-4.698135	0.838287
3	6	C	-2.191347	-5.718320	1.162943
4	7	N	-0.873355	-5.546955	0.997724
5	6	C	-0.434657	-4.324187	0.719976
6	6	C	0.925667	-4.099153	0.370446
7	6	C	1.338724	-2.881094	-0.096581
8	6	C	0.385075	-1.826964	-0.301059
9	6	C	-0.903091	-1.958076	0.098049
10	6	C	-1.359190	-3.158291	0.834027
11	1	H	-2.530979	-6.723489	1.380646
12	1	H	1.608522	-4.939089	0.441017
13	1	H	2.371485	-2.727187	-0.387069
14	1	H	0.702657	-0.923074	-0.810305
15	1	H	-1.636701	-1.178076	-0.074140
16	6	C	-4.562314	-5.015090	0.689162
17	6	C	-5.341317	-4.323201	-0.251289
18	6	C	-6.697707	-4.603615	-0.395806
19	6	C	-7.304989	-5.573455	0.403605
20	6	C	-6.542184	-6.261649	1.347722
21	6	C	-5.183382	-5.987152	1.488695
22	1	H	-4.867512	-3.573251	-0.872344
23	1	H	-7.281306	-4.067544	-1.136172
24	1	H	-8.361387	-5.790197	0.292489
25	1	H	-7.006074	-7.010830	1.979540
26	1	H	-4.609459	-6.519232	2.238278
27	3	Li	-3.352115	-2.291269	2.131139
28	6	C	-1.200028	-2.697189	2.549723
29	6	C	-0.309548	-2.491082	3.373359
30	6	C	0.709345	-2.292954	4.342894
31	6	C	1.226201	-1.007150	4.601654
32	6	C	2.232541	-0.828429	5.544797
33	6	C	2.745419	-1.922726	6.245006
34	6	C	2.242455	-3.201776	5.996788
35	6	C	1.232919	-3.389327	5.058943
36	1	H	0.832275	-0.159022	4.054159
37	1	H	2.620802	0.166175	5.733377
38	1	H	3.531262	-1.779890	6.977842
39	1	H	2.638113	-4.054703	6.536708
40	1	H	0.842003	-4.380998	4.864554

Table S14. Cartesian coordinates for the intermediate along RP-C10 (**5d**). The structure was optimized at the RB3LYP-GD3/6-311++G(d,p)/THF level of theory at 195.15 K. Coordinate values are given in Å.

Centre number	Atomic number	Atom type	Coordinates		
			X	Y	Z
1	7	N	-2.336929	-3.343725	0.646652
2	6	C	-2.679257	-4.616418	0.843093
3	6	C	-1.766293	-5.686588	0.753526
4	7	N	-0.587056	-5.522462	0.115457
5	6	C	-0.178105	-4.294610	-0.133240
6	6	C	0.879214	-4.047277	-1.053973
7	6	C	1.162927	-2.788093	-1.502913
8	6	C	0.347092	-1.676793	-1.079892
9	6	C	-0.620157	-1.812172	-0.147827
10	6	C	-0.866369	-3.118154	0.585436
11	1	H	-2.092984	-6.702072	0.941529
12	1	H	1.415876	-4.912562	-1.430483
13	1	H	1.948875	-2.624955	-2.230845
14	1	H	0.504770	-0.708899	-1.546285
15	1	H	-1.213607	-0.959097	0.165237
16	6	C	-4.124534	-4.930650	1.021559
17	6	C	-5.089586	-4.400625	0.151251
18	6	C	-6.440718	-4.699402	0.319711
19	6	C	-6.851893	-5.531084	1.362408
20	6	C	-5.899648	-6.065524	2.231617
21	6	C	-4.548233	-5.768914	2.063193
22	1	H	-4.768380	-3.776526	-0.675332
23	1	H	-7.171624	-4.292315	-0.370466
24	1	H	-7.902516	-5.763872	1.493664
25	1	H	-6.209906	-6.711145	3.045865
26	1	H	-3.814364	-6.180302	2.747223
27	3	Li	-3.546399	-1.867126	1.171405
28	6	C	-0.331745	-2.995968	1.968191
29	6	C	0.082852	-2.789215	3.086538
30	6	C	0.576359	-2.539118	4.402020
31	6	C	1.598736	-1.594950	4.611897
32	6	C	2.070958	-1.341054	5.896088
33	6	C	1.534549	-2.020441	6.991277
34	6	C	0.521583	-2.960351	6.793553
35	6	C	0.044767	-3.220932	5.512108
36	1	H	2.015355	-1.067865	3.761722
37	1	H	2.859482	-0.611460	6.042465
38	1	H	1.904114	-1.820150	7.990427
39	1	H	0.102856	-3.493320	7.639763
40	1	H	-0.739332	-3.952939	5.358696

Table S15. Cartesian coordinates for **6a**. The structure was optimized at the RB3LYP-GD3/6-311++G(d,p)/THF level of theory at 298.15 K. Coordinate values are given in Å.

Centre number	Atomic number	Atom type	Coordinates		
			X	Y	Z
1	7	N	-2.884519	-4.459438	-0.648736
2	6	C	-4.162593	-5.158049	-0.419877
3	6	C	-3.937555	-6.439546	0.406271
4	7	N	-3.021978	-6.548046	1.291193
5	6	C	-2.116788	-5.491112	1.433453
6	6	C	-1.217349	-5.502515	2.505923
7	6	C	-0.191036	-4.569879	2.594678
8	6	C	-0.041013	-3.637042	1.553101
9	6	C	-0.914461	-3.613787	0.477968
10	6	C	-2.018337	-4.509183	0.392724
11	1	H	-4.600896	-7.282705	0.220062
12	1	H	-1.337102	-6.274415	3.260073
13	1	H	0.492670	-4.575618	3.435280
14	1	H	0.769307	-2.915328	1.591529
15	1	H	-0.781462	-2.874140	-0.306484
16	6	C	-4.792570	-5.546493	-1.766409
17	6	C	-4.005652	-6.245554	-2.690564
18	6	C	-4.529959	-6.636636	-3.920657
19	6	C	-5.854512	-6.337366	-4.246318
20	6	C	-6.646974	-5.650789	-3.328629
21	6	C	-6.119014	-5.260737	-2.095746
22	1	H	-2.976872	-6.473666	-2.438423
23	1	H	-3.906139	-7.177099	-4.624193
24	1	H	-6.263594	-6.639807	-5.203808
25	1	H	-7.678101	-5.415886	-3.569005
26	1	H	-6.743867	-4.727930	-1.388876
27	3	Li	-2.919670	-3.126225	-2.105139
28	6	C	-5.067160	-4.284437	0.366136
29	6	C	-5.711676	-3.524087	1.053779
30	6	C	-6.467159	-2.640623	1.881817
31	6	C	-5.955305	-2.222852	3.123707
32	6	C	-6.692738	-1.363322	3.932097
33	6	C	-7.945067	-0.906869	3.517186
34	6	C	-8.459842	-1.315792	2.285765
35	6	C	-7.729992	-2.176574	1.471688
36	1	H	-4.983119	-2.578583	3.443048
37	1	H	-6.289461	-1.049374	4.888067
38	1	H	-8.516641	-0.237650	4.149900
39	1	H	-9.432607	-0.965203	1.960218
40	1	H	-8.130639	-2.498135	0.517435
41	8	O	-4.451174	-1.988473	-1.669071
42	1	H	-4.880730	-2.380244	-0.890263
43	1	H	-5.152472	-1.595542	-2.199239

Table S16. Cartesian coordinates for **7a**. The structure was optimized at the RB3LYP-GD3/6-311++G(d,p)/THF level of theory at 298.15 K. Coordinate values are given in Å.

Centre number	Atomic number	Atom type	Coordinates		
			X	Y	Z
1	7	N	-2.994156	-4.371609	-0.63414
2	6	C	-4.260137	-5.076676	-0.36131
3	6	C	-3.972621	-6.346619	0.460134
4	7	N	-3.010810	-6.449578	1.292705
5	6	C	-2.108933	-5.378166	1.398604
6	6	C	-1.167314	-5.377457	2.432743
7	6	C	-0.185302	-4.395209	2.50337
8	6	C	-0.130901	-3.418126	1.498719
9	6	C	-1.052892	-3.406367	0.459997
10	6	C	-2.084324	-4.370379	0.394967
11	1	H	-4.656479	-7.184905	0.336866
12	1	H	-1.220396	-6.169832	3.172258
13	1	H	0.533724	-4.392776	3.313973
14	1	H	0.635970	-2.651042	1.534766
15	1	H	-1.013264	-2.634677	-0.30132
16	6	C	-4.904315	-5.438288	-1.70742
17	6	C	-4.382985	-6.506764	-2.44767
18	6	C	-4.895816	-6.820026	-3.70545
19	6	C	-5.937120	-6.063196	-4.24517
20	6	C	-6.461822	-4.998505	-3.51445
21	6	C	-5.951266	-4.691963	-2.2521
22	1	H	-3.566854	-7.094205	-2.04126
23	1	H	-4.485248	-7.655895	-4.26096
24	1	H	-6.337086	-6.304698	-5.22346
25	1	H	-7.271617	-4.404951	-3.92396
26	1	H	-6.367548	-3.865884	-1.68818
27	3	Li	-2.344608	-4.015350	-2.9261
28	6	C	-5.163949	-4.245115	0.462315
29	6	C	-5.847624	-3.555249	1.181691
30	6	C	-6.659141	-2.737837	2.02261
31	6	C	-6.106599	-2.106798	3.151965
32	6	C	-6.901745	-1.309690	3.969806
33	6	C	-8.254786	-1.129183	3.677495
34	6	C	-8.811547	-1.751961	2.55902
35	6	C	-8.023650	-2.550429	1.73569
36	1	H	-5.056411	-2.248468	3.377393
37	1	H	-6.464701	-0.828747	4.837506
38	1	H	-8.871291	-0.508131	4.317172
39	1	H	-9.861999	-1.615824	2.328073
40	1	H	-8.455213	-3.035616	0.868189
41	8	O	-3.095803	-2.446010	-2.1847
42	1	H	-3.147679	-3.246023	-1.33868
43	1	H	-3.949567	-2.018531	-2.29003

Table S17. Cartesian coordinates for **8a**. The structure was optimized at the RB3LYP-GD3/6-311++G(d,p)/THF level of theory at 298.15 K. Coordinate values are given in Å.

Centre number	Atomic number	Atom type	Coordinates		
			X	Y	Z
1	7	N	-2.972067	-4.438597	-0.57498
2	6	C	-4.274519	-5.071644	-0.36078
3	6	C	-4.040779	-6.341081	0.48739
4	7	N	-3.087787	-6.483281	1.32242
5	6	C	-2.142543	-5.449938	1.445386
6	6	C	-1.217117	-5.483109	2.491778
7	6	C	-0.225443	-4.512212	2.594118
8	6	C	-0.148481	-3.510250	1.618447
9	6	C	-1.056497	-3.463781	0.56614
10	6	C	-2.078210	-4.424929	0.470233
11	1	H	-4.769907	-7.140423	0.370625
12	1	H	-1.291509	-6.287464	3.215613
13	1	H	0.484118	-4.537735	3.412463
14	1	H	0.626136	-2.753472	1.682779
15	1	H	-0.996314	-2.686009	-0.18693
16	6	C	-4.869381	-5.471208	-1.7185
17	6	C	-4.074493	-6.211587	-2.60226
18	6	C	-4.574121	-6.609758	-3.84024
19	6	C	-5.878217	-6.273940	-4.21153
20	6	C	-6.675617	-5.543520	-3.3328
21	6	C	-6.174185	-5.146661	-2.09093
22	1	H	-3.060188	-6.464519	-2.31775
23	1	H	-3.947687	-7.184548	-4.51334
24	1	H	-6.268201	-6.583510	-5.17463
25	1	H	-7.689888	-5.278951	-3.61019
26	1	H	-6.797842	-4.574280	-1.41529
27	3	Li	-3.599379	-3.040074	-3.84593
28	6	C	-5.181552	-4.205757	0.418887
29	6	C	-5.857623	-3.498546	1.127734
30	6	C	-6.652295	-2.660226	1.963828
31	6	C	-6.112761	-2.120517	3.1456
32	6	C	-6.889603	-1.304518	3.962157
33	6	C	-8.210873	-1.015568	3.616566
34	6	C	-8.754083	-1.547297	2.445684
35	6	C	-7.984101	-2.363214	1.622559
36	1	H	-5.087393	-2.346979	3.412258
37	1	H	-6.463373	-0.894052	4.870432
38	1	H	-8.813438	-0.380404	4.255636
39	1	H	-9.779774	-1.325926	2.173419
40	1	H	-8.404551	-2.776271	0.713322
41	8	O	-3.139076	-2.336963	-2.30026
42	1	H	-3.003930	-3.592576	-1.19911
43	1	H	-3.131274	-1.481455	-1.86836

Table S18. Cartesian coordinates for **6b**. The structure was optimized at the RB3LYP-GD3/6-311++G(d,p)/THF level of theory at 298.15 K. Coordinate values are given in Å.

Centre number	Atomic number	Atom type	Coordinates		
			X	Y	Z
1	7	N	-3.083964	-5.433416	0.533188
2	6	C	-3.622491	-5.685783	-0.612348
3	6	C	-2.985946	-6.726315	-1.545726
4	7	N	-1.520507	-6.698161	-1.471600
5	6	C	-1.044177	-6.546956	-0.214181
6	6	C	0.304060	-6.858834	0.120612
7	6	C	0.776092	-6.735570	1.416279
8	6	C	-0.052142	-6.262125	2.451614
9	6	C	-1.349738	-5.878548	2.142970
10	6	C	-1.854373	-6.000715	0.839141
11	1	H	-3.273604	-6.495401	-2.576193
12	1	H	0.958859	-7.238710	-0.657921
13	1	H	1.801733	-7.016500	1.635371
14	1	H	0.324949	-6.176750	3.463951
15	1	H	-2.002724	-5.457086	2.900875
16	6	C	-4.865858	-4.994116	-1.010076
17	6	C	-5.277383	-3.829965	-0.334314
18	6	C	-6.455193	-3.180697	-0.682445
19	6	C	-7.256433	-3.676591	-1.715537
20	6	C	-6.864490	-4.830147	-2.392161
21	6	C	-5.681251	-5.481743	-2.045633
22	1	H	-4.654251	-3.446403	0.463599
23	1	H	-6.750415	-2.281897	-0.152039
24	1	H	-8.174969	-3.168705	-1.987190
25	1	H	-7.482148	-5.229232	-3.189152
26	1	H	-5.411479	-6.388677	-2.572749
27	3	Li	-0.608203	-7.460386	-3.036403
28	6	C	-3.518970	-8.076517	-1.243007
29	6	C	-3.870839	-9.209736	-0.999906
30	6	C	-4.276532	-10.541667	-0.682742
31	6	C	-5.295793	-11.177749	-1.413813
32	6	C	-5.679092	-12.478039	-1.098767
33	6	C	-5.054204	-13.162936	-0.055227
34	6	C	-4.041970	-12.538816	0.675888
35	6	C	-3.653207	-11.238703	0.368378
36	1	H	-5.782305	-10.644598	-2.222100
37	1	H	-6.467790	-12.957167	-1.667643
38	1	H	-5.355216	-14.175533	0.187589
39	1	H	-3.554950	-13.065489	1.488667
40	1	H	-2.869313	-10.751533	0.935821
41	8	O	-1.563171	-9.118956	-3.400642
42	1	H	-1.695537	-9.758612	-4.107350
43	1	H	-2.272697	-9.257492	-2.751295

Table S19. Cartesian coordinates for **7b**. The structure was optimized at the RB3LYP-GD3/6-311++G(d,p)/THF level of theory at 298.15 K. Coordinate values are given in Å.

Centre number	Atomic number	Atom type	Coordinates		
			X	Y	Z
1	7	N	-3.054269	-5.590383	0.433896
2	6	C	-3.642666	-5.854378	-0.682147
3	6	C	-3.064154	-6.924250	-1.624008
4	7	N	-1.602778	-6.856777	-1.600250
5	6	C	-1.065490	-6.775662	-0.338609
6	6	C	0.254429	-7.185903	-0.055383
7	6	C	0.767107	-7.082881	1.231437
8	6	C	-0.005430	-6.551814	2.276018
9	6	C	-1.289357	-6.095216	2.004312
10	6	C	-1.828247	-6.192825	0.713969
11	1	H	-3.384258	-6.715663	-2.648081
12	1	H	0.850633	-7.612409	-0.855175
13	1	H	1.776013	-7.429163	1.430519
14	1	H	0.400296	-6.484226	3.278610
15	1	H	-1.898108	-5.642008	2.779742
16	6	C	-4.887813	-5.145585	-1.047386
17	6	C	-5.244492	-3.955581	-0.387717
18	6	C	-6.417039	-3.284980	-0.713681
19	6	C	-7.265197	-3.788070	-1.704426
20	6	C	-6.927600	-4.969280	-2.362512
21	6	C	-5.749077	-5.640763	-2.040451
22	1	H	-4.585065	-3.568320	0.378581
23	1	H	-6.671556	-2.365436	-0.198333
24	1	H	-8.179460	-3.263721	-1.958400
25	1	H	-7.583118	-5.373310	-3.125749
26	1	H	-5.520202	-6.567542	-2.552113
27	3	Li	-0.567135	-5.841404	-3.326121
28	6	C	-3.589049	-8.254278	-1.256962
29	6	C	-3.993358	-9.348959	-0.941856
30	6	C	-4.467530	-10.638126	-0.556032
31	6	C	-5.335037	-11.364245	-1.392193
32	6	C	-5.794190	-12.620592	-1.008167
33	6	C	-5.397019	-13.173204	0.210608
34	6	C	-4.535891	-12.460299	1.046620
35	6	C	-4.073300	-11.202816	0.670835
36	1	H	-5.642971	-10.935440	-2.338504
37	1	H	-6.463019	-13.169621	-1.661314
38	1	H	-5.756104	-14.152182	0.506683
39	1	H	-4.224328	-12.884432	1.994422
40	1	H	-3.405775	-10.648003	1.319219
41	8	O	-0.350857	-7.717645	-3.529016
42	1	H	-0.658213	-8.478100	-4.027217
43	1	H	-0.986959	-7.527480	-2.557881

Table S20. Cartesian coordinates for **8b**. The structure was optimized at the RB3LYP-GD3/6-311++G(d,p)/THF level of theory at 298.15 K. Coordinate values are given in Å.

Centre number	Atomic number	Atom type	Coordinates		
			X	Y	Z
1	7	N	-3.068024	-5.524598	0.452974
2	6	C	-3.671659	-5.790132	-0.652735
3	6	C	-3.095044	-6.831066	-1.632246
4	7	N	-1.640623	-6.743575	-1.580792
5	6	C	-1.078387	-6.705423	-0.325692
6	6	C	0.220286	-7.168885	-0.057220
7	6	C	0.742868	-7.072811	1.227999
8	6	C	-0.005310	-6.506585	2.269341
9	6	C	-1.279810	-6.017153	2.004810
10	6	C	-1.828516	-6.109014	0.719746
11	1	H	-3.397920	-6.581536	-2.651059
12	1	H	0.791602	-7.616261	-0.862907
13	1	H	1.740919	-7.449746	1.424712
14	1	H	0.409090	-6.441226	3.268421
15	1	H	-1.873576	-5.549398	2.782654
16	6	C	-4.952468	-5.124715	-0.972744
17	6	C	-5.344605	-3.975800	-0.262576
18	6	C	-6.549139	-3.343155	-0.543228
19	6	C	-7.394297	-3.845119	-1.536956
20	6	C	-7.020752	-4.985632	-2.245149
21	6	C	-5.809457	-5.618276	-1.969724
22	1	H	-4.687523	-3.589410	0.505988
23	1	H	-6.831228	-2.454335	0.010127
24	1	H	-8.334144	-3.350731	-1.755083
25	1	H	-7.672631	-5.387786	-3.012361
26	1	H	-5.552216	-6.513275	-2.522502
27	3	Li	0.397293	-6.707008	-4.519836
28	6	C	-3.617536	-8.171409	-1.303512
29	6	C	-4.010129	-9.272856	-0.999154
30	6	C	-4.468943	-10.570178	-0.623158
31	6	C	-5.260032	-11.335831	-1.498583
32	6	C	-5.702282	-12.600314	-1.121359
33	6	C	-5.363290	-13.121090	0.128566
34	6	C	-4.577898	-12.368410	1.003380
35	6	C	-4.132985	-11.102534	0.634972
36	1	H	-5.521506	-10.931843	-2.469416
37	1	H	-6.311845	-13.180788	-1.804565
38	1	H	-5.708863	-14.106651	0.418806
39	1	H	-4.311859	-12.767999	1.975378
40	1	H	-3.523990	-10.516553	1.312897
41	8	O	-0.286433	-8.017609	-3.570594
42	1	H	-0.339488	-8.973836	-3.541496
43	1	H	-1.135280	-7.292417	-2.320337

Table S21. Cartesian coordinates for **7e**. The structure was optimized at the RB3LYP-GD3/6-311++G(d,p)/THF level of theory at 298.15 K. Coordinate values are given in Å.

Centre number	Atomic number	Atom type	Coordinates		
			X	Y	Z
1	7	N	-2.827494	-3.297539	-0.329438
2	6	C	-3.698411	-3.855217	0.546387
3	6	C	-3.203095	-4.823379	1.528126
4	7	N	-2.026157	-5.347885	1.478892
5	6	C	-1.201729	-4.950278	0.434644
6	6	C	0.054892	-5.557903	0.284301
7	6	C	0.920622	-5.151340	-0.717857
8	6	C	0.542799	-4.102652	-1.575964
9	6	C	-0.688419	-3.485544	-1.441018
10	6	C	-1.604069	-3.907128	-0.448774
11	1	H	-3.873228	-5.119900	2.330205
12	1	H	0.324208	-6.349438	0.974787
13	1	H	1.886490	-5.628465	-0.834065
14	1	H	1.224374	-3.771516	-2.351875
15	1	H	-0.965440	-2.671467	-2.103195
16	6	C	-4.896589	-5.511925	-0.505234
17	6	C	-4.083773	-6.371844	-1.258452
18	6	C	-4.340219	-7.744606	-1.323325
19	6	C	-5.418148	-8.284206	-0.615710
20	6	C	-6.235356	-7.445906	0.147204
21	6	C	-5.965046	-6.074748	0.203485
22	1	H	-3.234880	-5.967816	-1.806226
23	1	H	-3.702872	-8.394443	-1.916500
24	1	H	-5.616469	-9.350171	-0.654867
25	1	H	-7.074678	-7.862842	0.697001
26	1	H	-6.599975	-5.434474	0.814173
27	3	Li	-3.761251	-2.182751	-1.750767
28	6	C	-4.853200	-3.096109	0.921838
29	6	C	-5.824976	-2.467444	1.271983
30	6	C	-6.982744	-1.731604	1.650511
31	6	C	-7.664343	-2.032147	2.844682
32	6	C	-8.798614	-1.312115	3.205584
33	6	C	-9.271584	-0.284125	2.387810
34	6	C	-8.601954	0.020788	1.201485
35	6	C	-7.467161	-0.694021	0.831827
36	1	H	-7.297263	-2.829841	3.479365
37	1	H	-9.314904	-1.553361	4.127752
38	1	H	-10.155566	0.274669	2.672591
39	1	H	-8.965672	0.816833	0.561719
40	1	H	-6.950129	-0.459827	-0.091043
41	8	O	-5.263641	-3.278969	-2.134881
42	1	H	-5.189517	-4.067714	-1.489849
43	1	H	-5.614226	-3.640056	-2.954535

Table S22. Cartesian coordinates for **8e**. The structure was optimized at the RB3LYP-GD3/6-311++G(d,p)/THF level of theory at 298.15 K. Coordinate values are given in Å.

Centre number	Atomic number	Atom type	Coordinates		
			X	Y	Z
1	7	N	-2.941655	-4.142266	0.652447
2	6	C	-3.857871	-4.766341	1.387004
3	6	C	-3.675737	-6.124264	1.803735
4	7	N	-2.623605	-6.825811	1.476055
5	6	C	-1.683129	-6.215143	0.697297
6	6	C	-0.542695	-6.940407	0.281366
7	6	C	0.405936	-6.336239	-0.510920
8	6	C	0.253215	-4.986379	-0.911786
9	6	C	-0.844068	-4.256340	-0.520450
10	6	C	-1.839549	-4.855076	0.289053
11	1	H	-4.439536	-6.596777	2.413100
12	1	H	-0.446109	-7.971207	0.600517
13	1	H	1.278026	-6.892566	-0.833332
14	1	H	1.010176	-4.527765	-1.537045
15	1	H	-0.971412	-3.227118	-0.833188
16	6	C	-5.258434	-3.999146	-2.139965
17	6	C	-4.026505	-4.419754	-2.643017
18	6	C	-3.587303	-5.724273	-2.415416
19	6	C	-4.380377	-6.607909	-1.683274
20	6	C	-5.614599	-6.188359	-1.183251
21	6	C	-6.053704	-4.884290	-1.411515
22	1	H	-3.404776	-3.728023	-3.200616
23	1	H	-2.622332	-6.045670	-2.790781
24	1	H	-4.032875	-7.617045	-1.492727
25	1	H	-6.226133	-6.873405	-0.606443
26	1	H	-7.003011	-4.552215	-1.007505
27	3	Li	-3.449957	-2.123527	0.117835
28	6	C	-5.033391	-4.052199	1.723795
29	6	C	-6.019706	-3.373254	1.900585
30	6	C	-7.144015	-2.516922	2.028819
31	6	C	-8.283019	-2.909108	2.755522
32	6	C	-9.373939	-2.052028	2.848222
33	6	C	-9.340982	-0.804251	2.221331
34	6	C	-8.210988	-0.411985	1.500030
35	6	C	-7.110516	-1.256639	1.397745
36	1	H	-8.302526	-3.878733	3.238659
37	1	H	-10.250462	-2.355902	3.408639
38	1	H	-10.194278	-0.139714	2.295843
39	1	H	-8.187601	0.557281	1.014929
40	1	H	-6.221881	-0.963181	0.841050
41	8	O	-4.133617	-0.580662	-0.188365
42	1	H	-5.591490	-2.980400	-2.303497
43	1	H	-4.289380	0.328257	-0.438824

Table S23. Energetic information for the reactants (**1** and **2**). The structures were optimized at the RB3LYP-GD3/6-311++G(d,p)/THF level of theory at 195.15 K and 1 atm. Energy values are given in Hartree (a.u.) units.

	**REACTANTS**	
	**1**	**2**
Electronic Energy (EE)	-649.2102	-315.4706
Zero-point Energy Correction	0.2036	0.0995
Thermal Correction to Energy	0.2087	0.1032
Thermal Correction to Enthalpy	0.2093	0.1039
Thermal Correction to Free Energy	0.1814	0.0806
EE + Zero-point Energy	-649.0067	-315.3710
EE + Thermal Energy Correction	-649.0016	-315.3673
EE + Thermal Enthalpy Correction	-649.0010	-315.3667
EE + Thermal Free Energy Correction	-649.0288	-315.3900

Table S24. Energetic information for all the stationary points involved in the nucleophilic addition step along RP-C2. The structures were optimized at the RB3LYP-GD3/6-311++G(d,p)/THF level of theory at 195.15 K and 1 atm. Energy values are given in Hartree (a.u.) units.

	**RP-C2**		
	**3a**	**4a**	**5a**
Electronic Energy (EE)	-964.6927	-964.6518	-964.6811
Zero-point Energy Correction	0.3054	0.3031	0.3050
Thermal Correction to Energy	0.3149	0.3122	0.3141
Thermal Correction to Enthalpy	0.3155	0.3129	0.3147
Thermal Correction to Free Energy	0.2768	0.2744	0.2767
EE + Zero-point Energy	-964.3873	-964.3488	-964.3760
EE + Thermal Energy Correction	-964.3778	-964.3396	-964.3670
EE + Thermal Enthalpy Correction	-964.3772	-964.3390	-964.3663
EE + Thermal Free Energy Correction	-964.4159	-964.3775	-964.4044

Table S25. Energetic information for all the stationary points involved in the nucleophilic addition step along RP-C3. The structures were optimized at the RB3LYP-GD3/6-311++G(d,p)/THF level of theory at 195.15 K and 1 atm. Energy values are given in Hartree (a.u.) units.

	**RP-C3**		
	**3b**	**4b**	**5b**
Electronic Energy (EE)	-964.6974	-964.6620	-964.6911
Zero-point Energy Correction	0.3045	0.3040	0.3056
Thermal Correction to Energy	0.3145	0.3130	0.3148
Thermal Correction to Enthalpy	0.3151	0.3137	0.3154
Thermal Correction to Free Energy	0.2745	0.2754	0.2769
EE + Zero-point Energy	-964.3929	-964.3580	-964.3855
EE + Thermal Energy Correction	-964.3829	-964.3489	-964.3763
EE + Thermal Enthalpy Correction	-964.3823	-964.3483	-964.3757
EE + Thermal Free Energy Correction	-964.4229	-964.3865	-964.4142

Table S26. Energetic information for all the stationary points involved in the nucleophilic addition step along RP-C5. The structures were optimized at the RB3LYP-GD3/6-311++G(d,p)/THF level of theory at 195.15 K and 1 atm. Energy values are given in Hartree (a.u.) units.

	**RP-C5**		
	**3c**	**4c**	**5c**
Electronic Energy (EE)	-964.6943	-964.6337	-964.6585
Zero-point Energy Correction	0.3047	0.3027	0.3046
Thermal Correction to Energy	0.3145	0.3117	0.3137
Thermal Correction to Enthalpy	0.3151	0.3123	0.3144
Thermal Correction to Free Energy	0.2742	0.2742	0.2758
EE + Zero-point Energy	-964.3896	-964.3310	-964.3539
EE + Thermal Energy Correction	-964.3798	-964.3220	-964.3448
EE + Thermal Enthalpy Correction	-964.3792	-964.3214	-964.3441
EE + Thermal Free Energy Correction	-964.4201	-964.3595	-964.3827

Table S27. Energetic information for all the stationary points involved in the nucleophilic addition step along RP-C10. The structures were optimized at the RB3LYP-GD3/6-311++G(d,p)/THF level of theory at 195.15 K and 1 atm. Energy values are given in Hartree (a.u.) units.

	**RP-C10**		
	**3d**	**4d**	**5d**
Electronic Energy (EE)	-964.6939	-964.6331	-964.6531
Zero-point Energy Correction	0.3048	0.3025	0.3047
Thermal Correction to Energy	0.3146	0.3116	0.3138
Thermal Correction to Enthalpy	0.3152	0.3123	0.3144
Thermal Correction to Free Energy	0.2745	0.2741	0.2763
EE + Zero-point Energy	-964.3891	-964.3307	-964.3484
EE + Thermal Energy Correction	-964.3794	-964.3215	-964.3394
EE + Thermal Enthalpy Correction	-964.3788	-964.3209	-964.3387
EE + Thermal Free Energy Correction	-964.4194	-964.3590	-964.3769

Table S28. Energetic information for **H_2O**. This structure was optimized at the RB3LYP-GD3/6-311++G(d,p)/THF level of theory at 298.15 K and 1 atm. Energy values are given in Hartree (a.u.) units.

	**REACTANT**
	**H_2O**
Electronic Energy (EE)	-76.4650
Zero-point Energy Correction	0.0212
Thermal Correction to Energy	0.0241
Thermal Correction to Enthalpy	0.0250
Thermal Correction to Free Energy	0.0029
EE + Zero-point Energy	-76.4438
EE + Thermal Energy Correction	-76.4410
EE + Thermal Enthalpy Correction	-76.4400
EE + Thermal Free Energy Correction	-76.4621

Table S29. Energetic information for all the stationary points involved in the hydrolysis step along RP-C2. The structures were optimized at the RB3LYP-GD3/6-311++G(d,p)/THF level of theory at 298.15 K and 1 atm. Energy values are given in Hartree (a.u.) units.

	**RP-C2**		
	**6a**	**7a**	**8a**
Electronic Energy (EE)	-1041.1635	-1041.1582	-1041.1704
Zero-point Energy Correction	0.3289	0.3254	0.3296
Thermal Correction to Energy	0.3520	0.4744	0.3524
Thermal Correction to Enthalpy	0.3530	0.3484	0.3534
Thermal Correction to Free Energy	0.2738	0.2718	0.2751
EE + Zero-point Energy	-1040.8346	-1040.8328	-1040.8408
EE + Thermal Energy Correction	-1040.8115	-1040.8108	-1040.8180
EE + Thermal Enthalpy Correction	-1040.8106	-1040.8098	-1040.8171
EE + Thermal Free Energy Correction	-1040.8897	-1040.8864	-1040.8953

Table S30. Energetic information for all the stationary points involved in the hydrolysis step along RP-C3. These structures were optimized at the RB3LYP-GD3/6-311++G(d,p)/THF level of theory at 298.15 K and 1 atm. Energy values are given in Hartree (a.u.) units.

	**RP-C3**		
	**6b**	**7b**	**8b**
Electronic Energy (EE)	-1041.1732	-1041.1685	-1041.1795
Zero-point Energy Correction	0.3297	0.3258	0.3302
Thermal Correction to Energy	0.3529	0.3482	0.3533
Thermal Correction to Enthalpy	0.3538	0.3491	0.3542
Thermal Correction to Free Energy	0.2745	0.2712	0.2741
EE + Zero-point Energy	-1040.8435	-1040.8426	-1040.8493
EE + Thermal Energy Correction	-1040.8203	-1040.8203	-1040.8263
EE + Thermal Enthalpy Correction	-1040.8194	-1040.8193	-1040.8253
EE + Thermal Free Energy Correction	-1040.8987	-1040.8973	-1040.9054

Table S31. Energetic information for all the stationary points involved in the hydrolysis step along RP-C2. The structures were optimized at the RB3LYP-GD3/6-311++G(d,p)/THF level of theory at 298.15 K and 1 atm. Energy values are given in Hartree (a.u.) units.

	**RP-C2**		
	**6a**	**7e**	**8e**
Electronic Energy (EE)	-1041.1635	-1041.1219	-1041.1875
Zero-point Energy Correction	0.3289	0.3267	0.3282
Thermal Correction to Energy	0.3520	0.3492	0.3524
Thermal Correction to Enthalpy	0.3530	0.3502	0.3534
Thermal Correction to Free Energy	0.2738	0.2723	0.2702
EE + Zero-point Energy	-1040.8346	-1040.7952	-1040.8593
EE + Thermal Energy Correction	-1040.8115	-1040.7727	-1040.8351
EE + Thermal Enthalpy Correction	-1040.8106	-1040.7717	-1040.8342
EE + Thermal Free Energy Correction	-1040.8897	-1040.8496	-1040.9173

Part 2 – Selected geometric data

Table S32. Computed dihedral angles (DA) in º, angles in º and bond lengths in Å for energy optimised 2-phenylquinoxaline (**1**).

Energy optimized 2-phenylquinoxaline (**1**)

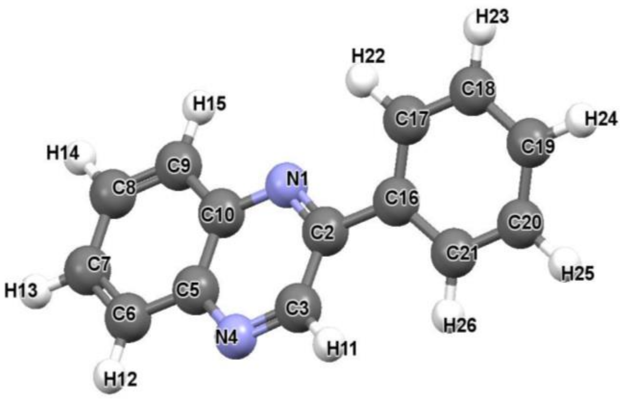

Dihedral angle (º)		Angle (º)		Distance (Å)	
C6,C5,C10,C9	0.10	N1,C2,C3	120.47	C3–H11	1.09
C6,C7,C8,C9	0.11	N1,C2,C16	118.56	C2–C16	1.48
N1,C2,C3,N4	1.19	C3,C2,C16	120.96	N1–C2	1.32
N4,C5,C10,N1	1.06	C2,C3,N4	123.12	C2–C3	1.43
N1,C2,C16,C17	28.37	N4,C3,C2	116.90	C3–N4	1.31
		H11,C3,C2	119.96	N4–C5	1.37
		N4,C5,C10	120.37	C5–C6	1.42
		N4,C5,C6	120.00	C5–C10	1.43
		C6,C5,C10	119.63	N1–C10	1.36
		C5,C10,N1	121.05	C10C9	1.42
		C5,C10,C9	119.22		
		C9,C10,N1	119.73		

Table S33. Computed dihedral angles (DA) in º, angles in º and bond lengths in Å for energy optimised lithium phenylacetylide (**2**).

Energy optimised lithium phenylacetylide (**2**)

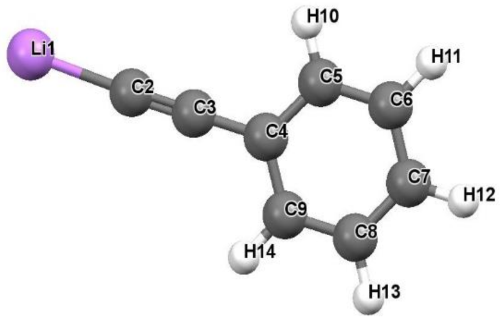

Dihedral angle (º)	Angle (º)		Distance (Å)	
	Li1,C2,C3	177.94	Li1–C2	2.04

Table S34. Computed dihedral angles (DA) in º, angles in º and bond lengths in Å for the energy optimised adduct **3a**, transition state **4a** and intermediate **5a** along RP-C2.

		RP-C2		
		**3a**	**4a**	**5a**
**Dihedral angle (º)**	C6,C5,C10,C9	0.92	1.37	3.79
	C6,C7,C8,C9	0.49	0.80	1.95
	N1,C2,C3,N4	–0.10	18.44	32.70
	N4,C5,C10,N1	1.61	3.68	8.72
	N1,C2,C16,C17	37.69	15.86	63.34
	N1,Li27,C28,C2		–17.21	
**Angle (º)**	Li27,C28,C29	137.54		
	N1,C2,C3	120.51	116.16	110.32
	N1,C2,C16	119.55	114.67	109.04
	C3,C2,C16	119.93	113.72	109.13
**Distance (Å)**	N1–C2	1.32	1.38	1.48
	C2–C3	1.43	1.49	1.54
	C2–C16	1.48	1.52	1.54
	Li27–C28	2.04	2.20	
	Li27–N1	2.23	2.01	1.95
	C28–C2	3.59	1.89	1.48

Energy optimized structures

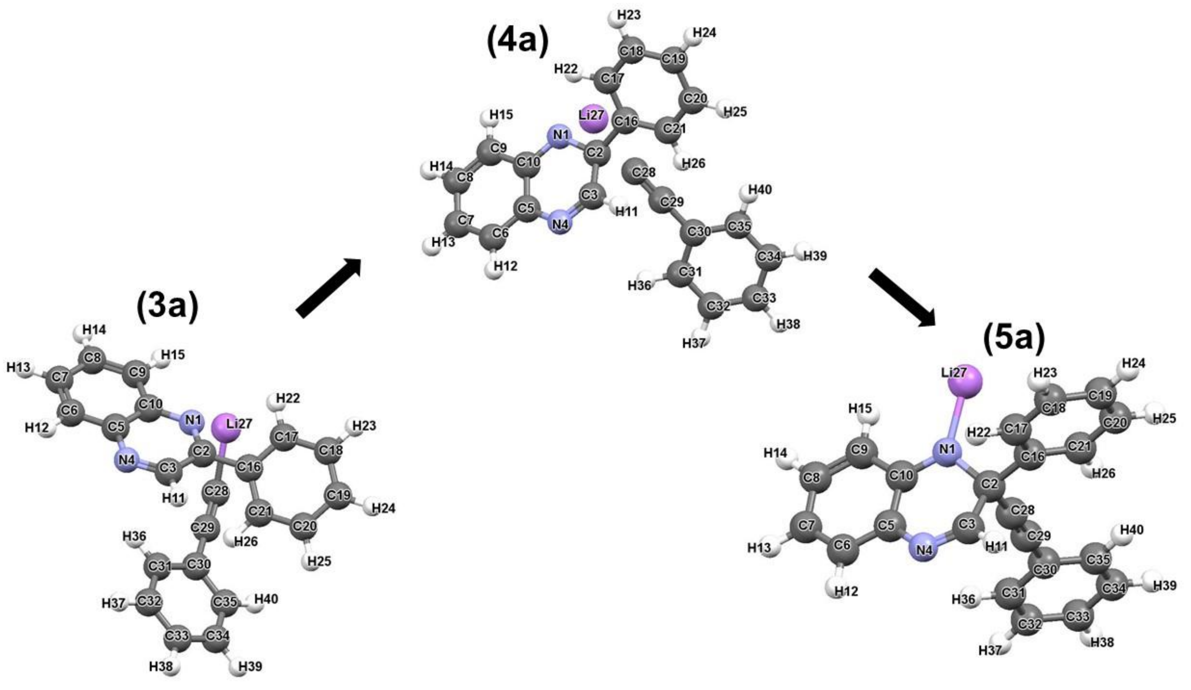

Table S35. Computed dihedral angles (DA) in º, angles in º and bond lengths in Å for the energy optimised adduct **3b**, transition state **4b** and intermediate **5b** along RP-C3.

		**RP-C3**		
		**3b**	**4b**	**5b**
**Dihedral angle (º)**	C6,C5,C10,C9	0.34	–1.59	–4.84
	C6,C7,C8,C9	0.18	–0.89	–2.21
	N1,C2,C3,N4	1.76	–20.31	–38.56
	N4,C5,C10,N1	1.32	–3.91	–10.42
	N1,C2,C16,C17	23.08	–5.53	18.25
	N4,Li27,C28,C3		10.53	
**Angle (º)**	Li27,C28,C29	155.49		
	C2,C3,N4	123.06	117.97	110.81
	C2,C3,H11	120.91	114.71	
	N4,C3,H11	116.03	112.25	
**Distance (Å)**	N4–C3	1.31	1.38	1.47
	C2–C3	1.43	1.50	1.54
	C3–H11	1.08	1.08	1.09
	Li27–C28	2.06	2.16	
	Li27–N4	2.15	2.02	1.94
	C28–C3	3.31	1.86	1.48

Energy optimized structures

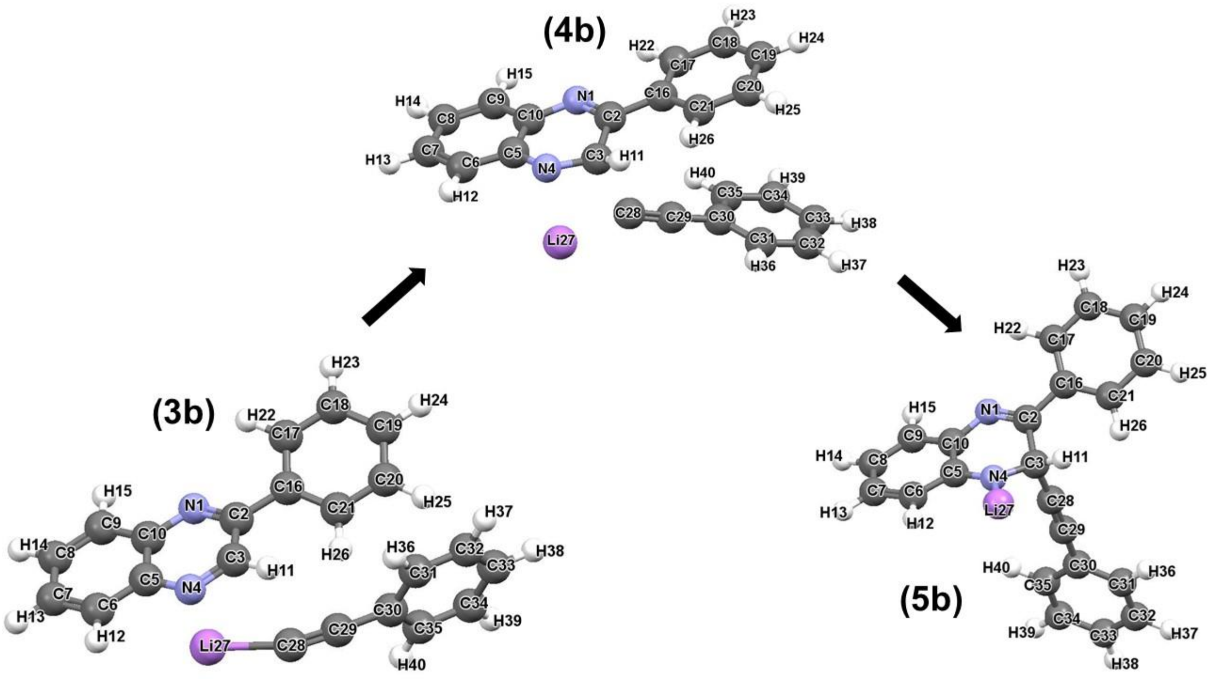

Table S36. Computed dihedral angles (DA) in º, angles in º and bond lengths in Å for the energy optimised adduct **3c**, transition state **4c** and intermediate **5c** along RP-C5.

		RP-C5		
		**3c**	**4c**	**5c**
**Dihedral angle (º)**	C6,C5,C10,C9	0.20	20.60	18.35
	C6,C7,C8,C9	0.13	7.06	6.08
	N1,C2,C3,N4	1.20	–14.34	–18.97
	N4,C5,C10,N1	1.09	–31.80	–42.50
	N1,C2,C16,C17	28.72	–16.95	–11.82
	N4,Li27,C28,C5		13.04	
**Angle (º)**	Li27,C28,C29	172.34		
	N4,C5,C10	119.63	114.21	108.73
	N4,C5,C6	120.45	112.19	108.53
	C6,C5,C10	119.92	104.14	111.53
**Distance (Å)**	N4–C5	1.37	1.43	1.49
	C5–C6	1.42	1.48	1.52
	C5–C10	1.43	1.49	1.54
	Li27–C28	2.04	2.21	
	Li27–N4	2.11	2.08	1.98
	C28–C5	4.19	1.79	1.49

Energy optimized structures

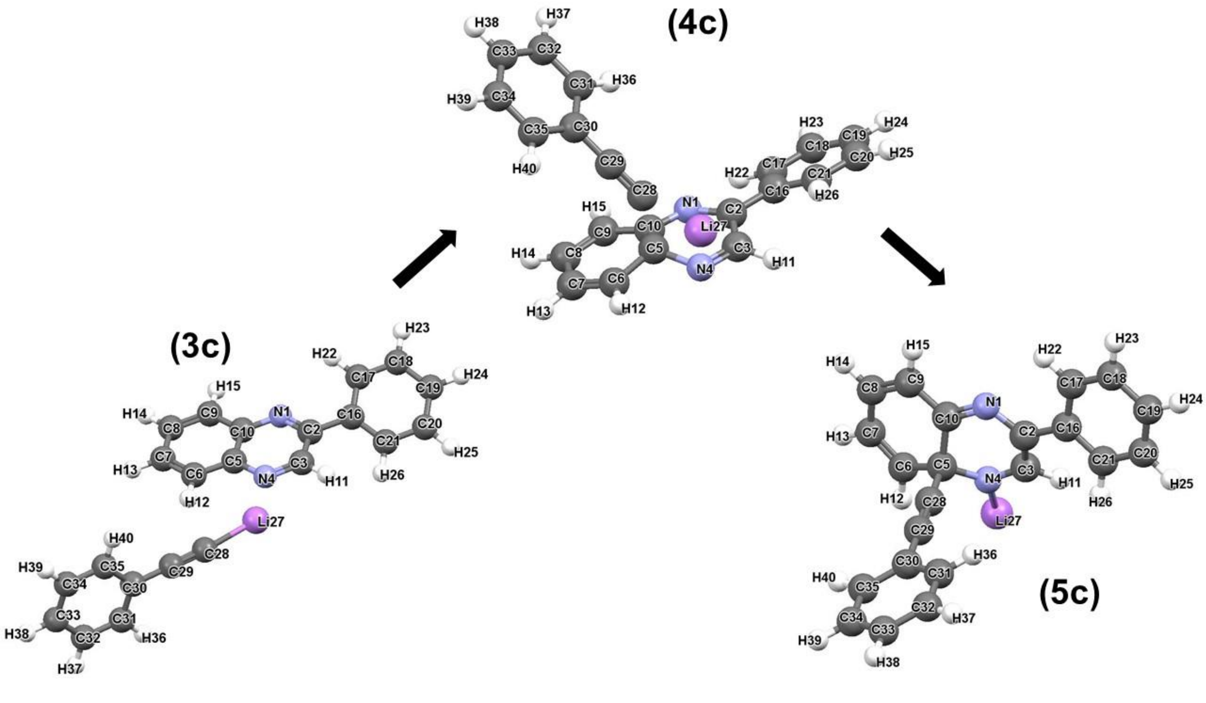

Table S37. Computed dihedral angles (DA) in º, angles in º and bond lengths in Å for the energy optimised adduct **3d**, transition state **4d** and intermediate **5d** along RP-C10.

		**RP-C10**		
		**3d**	**4d**	**5d**
**Dihedral angle (º)**	C6,C5,C10,C9	0.29	21.55	19.34
	C6,C7,C8,C9	0.20	7.43	6.46
	N1,C2,C3,N4	–0.51	-11.69	–18.08
	N4,C5,C10,N1	0.55	–30.36	–40.70
	N1,C2,C16,C17	44.21	29.94	47.14
	N1,Li27,C28,C10	15.20		
**Angle (º)**	Li27,C28,C29	175.62		
	N1,C10,C5	120.50	114.90	110.17
	N1,C10,C9	120.14	111.85	108.08
	C9,C10,C5	119.36	113.87	111.05
**Distance (Å)**	N1–C10	1.37	1.43	1.49
	C5–C10	1.43	1.49	1.54
	C9–C10	1.42	1.48	1.52
	Li27–C28	2.04	2.23	
	Li27–N1	2.15	2.04	1.98
	C28–C10	4.21	1.78	1.49

Energy optimized structures

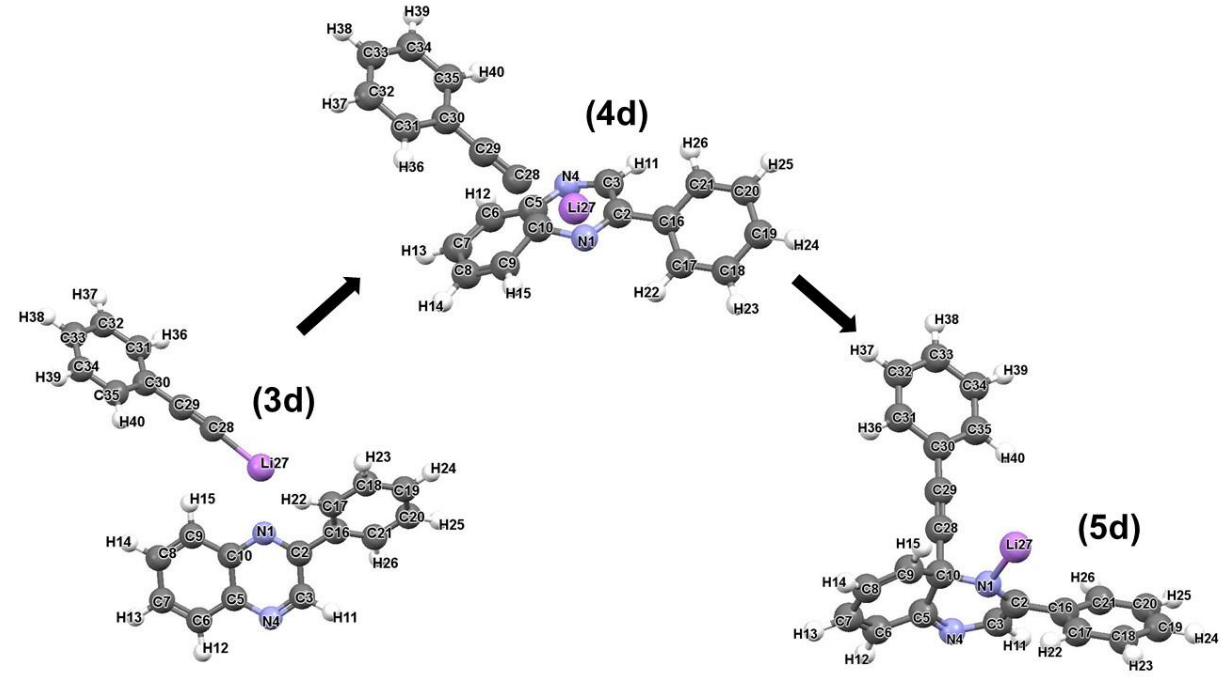

Table S38. Computed dihedral angles in º, angles in º and bond lengths in Å for the energy optimised stationary points **6a**, **7a** and **8a** along RP-C2.

		RP-C2		
		**6a**	**7a**	**8a**
**Dihedral angle (º)**	C6,C5,C10,C9	3.68	2.10	1.08
	C6,C7,C8,C9	1.89	1.24	0.89
	N1,C2,C3,N4	32.04	30.95	28.63
	N4,C5,C10,N1	8.75	4.68	1.59
	N1,C2,C16,C17	51.67	75.79	50.84
	N1,Li27,O41,H42	-4.64		
**Angle (º)**	N1,Li27,O41	104.01		
	N1,C2,C3	110.52	109.44	107.53
	N1,C2,C16	109.83	108.10	109.29
	C3,C2,C16	108.63	110.60	109.14
**Distance (Å)**	N1–C2	1.47	1.47	1.46
	C2–C3	1.54	1.54	1.54
	C2–C16	1.53	1.53	1.54
	Li27–O41	1.96	1.89	1.76
	Li27–N1	1.96	2.41	3.61
	O41–H42	0.97	1.16	1.66
	N1–H42	2.89	1.34	1.05

Energy optimized structures

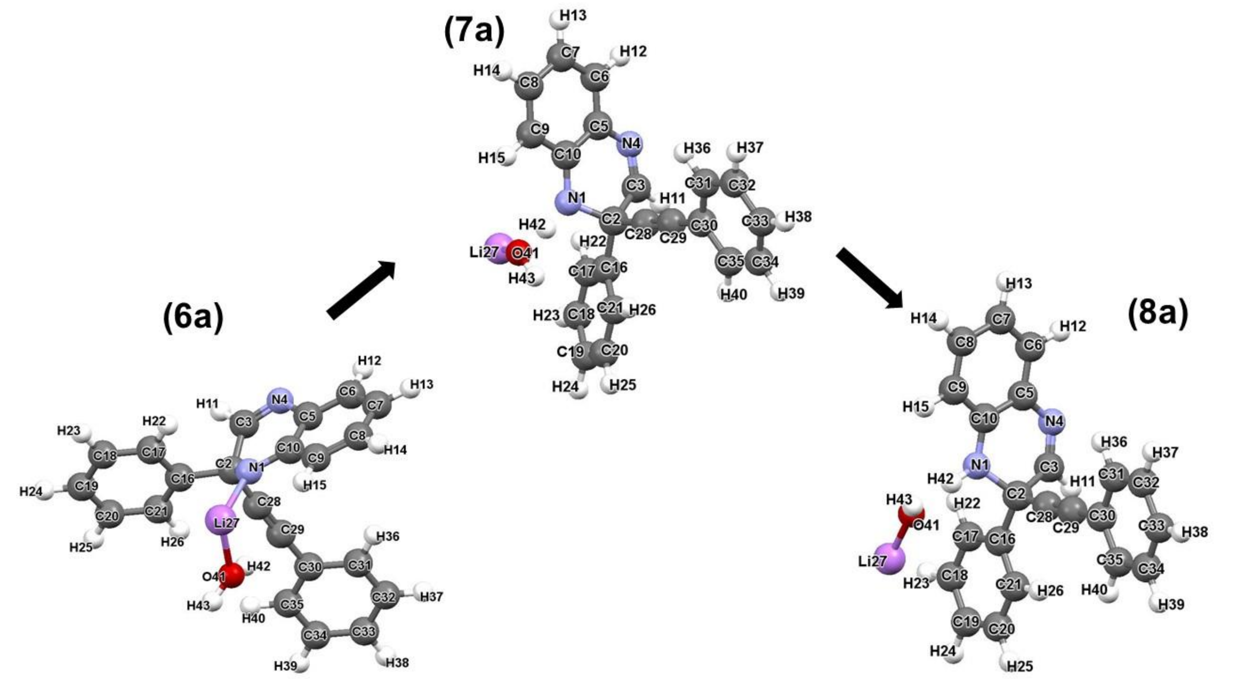

Table S39. Computed dihedral angles (DA) in º, angles in º and bond lengths in Å for the energy optimised stationary points **6b**, **7b** and **8b** along RP-C3.

		RP-C3		
		**6b**	**7b**	**8b**
**Dihedral angle (º)**	C6,C5,C10,C9	-4.32	-2.96	-1.40
	C6,C7,C8,C9	-1.95	-1.54	-0.97
	N1,C2,C3,N4	-36.18	-37.59	-35.18
	N4,C5,C10,N1	-9.53	-6.37	-3.10
	N1,C2,C16,C17	16.70	17.08	16.09
	N4,Li27,O41,H43	-1.08		
**Angle (º)**	N4,Li27,O41	104.57		
	N4,C3,C2	111.73	109.49	108.08
	N4,C3,H11	107.82	107.39	107.21
	C2,C3,H11	108.68	109.32	109.56
**Distance (Å)**	N4–C3	1.47	1.46	1.46
	C2–C3	1.54	1.54	1.54
	C3–H11	1.10	1.09	1.09
	Li27–O41	1.95	1.90	1.76
	Li27–N4	1.94	2.25	3.58
	O41–H43	0.97	1.17	1.68
	N4–H43	2.96	1.32	1.05

Energy optimized structures

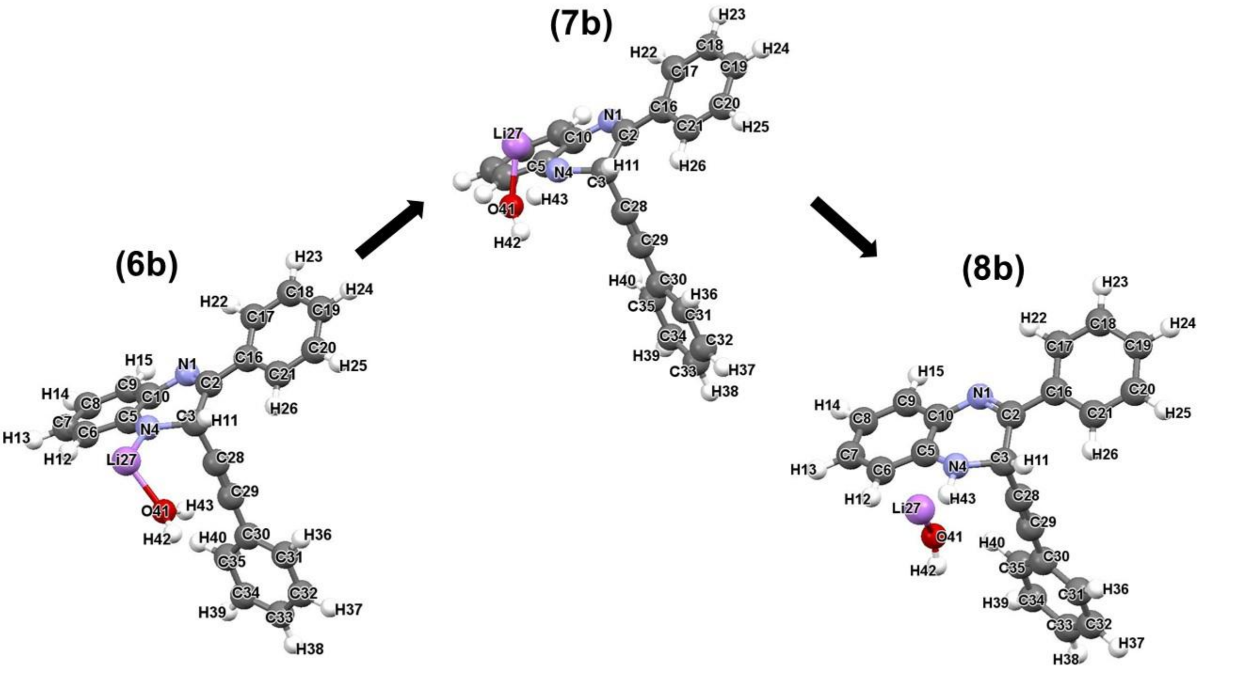

Table S40. Computed dihedral angles (DA) in º, angles in º and bond lengths in Å for the energy optimised stationary points **6a**, **7e** and **8e** along RP-C2.

		RP-C2		
		**6a**	**7e**	**8e**
**Dihedral angle (º)**	C6,C5,C10,C9	3.68	-1.87	-0.31
	C6,C7,C8,C9	1.89	-0.82	-0.13
	N1,C2,C3,N4	32.04	-11.68	-0.98
	N4,C5,C10,N1	8.75	-2.33	-0.96
	N1,C2,C16,C17	51.67		
	N1,Li27,O41,H42	-4.64		
**Angle (º)**	N1,Li27,O41	104.01	100.85	
	N1,C2,C3	110.52	119.19	121.20
	N1,C2,C16	109.83		
	C3,C2,C16	108.63		
**Distance (Å)**	N1–C2	1.47	1.36	1.33
	C2–C3	1.54	1.47	1.43
	C2–C16	1.54	2.30	3.87
	Li27–O41	1.96	1.90	1.72
	Li27–N1	1.98	2.03	2.15
	O41–H42	0.97	1.02	3.52
	C16–H42	3.29	1.77	1.08

Energy optimized structures

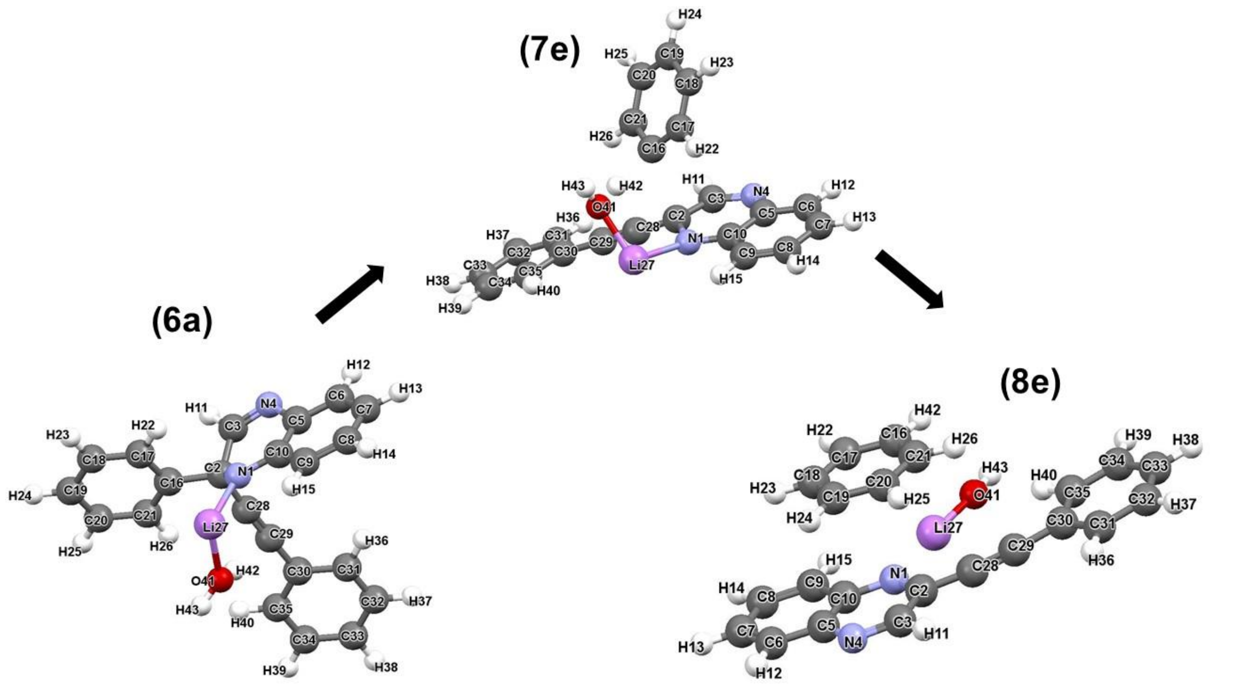

Table S41. Calculated energies (*E*, E_{ZPVE}, *H* and *G* in au) of adducts **3a**, **3b**, **3c** and **3d** as well as the relative energies (Δ) in kcal mol^{-1} denoting the energy difference between the computed energies of the adducts and the total sum of the energies of the reactants (**1** and **2**).

	E	E_{ZPVE}	*H*	*G*
	Reactants			
**1**	–649.2102	–649.0067	–649.0009	–649.0288
**2**	–315.4706	–315.3710	–315.3667	–315.3900
Total:	–964.6808	–964.3777	–964.3676	–964.4188
	Adducts			
**3a**	–964.6927	–964.3873	–964.3772	–964.4159
Δ	–7.5	–6.0	–6.0	1.8
**3b**	–964.6974	–964.3929	–964.3823	–964.4229
Δ	–10.4	–9.5	–9.2	–2.6
**3c**	–964.6943	–964.3896	–964.3792	–964.4201
Δ	–8.5	–7.5	–7.2	–0.8
**3d**	–964.6939	–964.3891	–964.3788	–964.4194
Δ	–8.2	–7.2	–7.0	–0.4

Part 3

Most attractive and repulsive diatomic interactions in 3, 4 and 5 along four reaction pathways considered

Table S42. Most significant (leading) attractive and repulsive diatomic inter-molecular interactions in adducts **3** formed between **1** and **2** along four potential reaction pathways (RP). All values in kcal/mol.

Atom A	Atom B	RP-C2	Atom A	Atom B	RP-C3	Atom A	Atom B	RP-C5	Atom A	Atom B	RP-C10
Attractive interactions with $\lvert E_{int}(A,B)\rvert$ > ~10 kcal/mol											
N1	Li27	-175.0	N4	Li27	-184.9	N4	Li27	-186.6	N1	Li27	-182.5
N4	Li27	-70.7	N1	Li27	-68.6	N1	Li27	-68.2	N4	Li27	-68.2
C2	C29	-33.9	C3	C29	-32.7	C3	C29	-22.8	C2	C29	-21.5
C3	C29	-28.1	C2	C29	-23.3	C5	C29	-19.3	C10	C29	-19.0
C10	C29	-23.6	C3	C28	-20.8	C3	C28	-18.9	C2	C28	-18.0
C5	C29	-19.6	C5	C29	-19.6	C2	C29	-16.9	C3	C29	-17.6
C2	C28	-16.3	C10	C29	-16.5	C5	C28	-15.4	C10	C28	-15.7
C10	C28	-12.9	C5	C28	-12.8	C10	C29	-14.9	C5	C29	-14.2
C3	C28	-9.7	H11	C29	-11.2	C2	C28	-11.7	C3	C28	-12.4
			C2	C28	-10.6				C5	C28	-9.6
									H15	C28	-9.2
Repulsive interactions with $\lvert E_{int}(A,B)\rvert$ > ~10 kcal/mol											
N4	C28	17.0	H11	Li27	11.1	N1	C28	21.6	N4	C28	21.8
C5	Li27	30.5	N1	C28	19.0	C10	Li27	30.9	C5	Li27	29.9
N1	C28	35.9	C10	Li27	30.3	N1	C29	32.9	N4	C29	32.6
C3	Li27	40.8	C2	Li27	38.1	C2	Li27	37.7	C3	Li27	39.5
N1	C29	49.6	N1	C29	40.9	N4	C28	47.1	N1	C28	47.1
C10	Li27	50.6	N4	C28	43.1	C5	Li27	50.3	C10	Li27	50.6
C2	Li27	63.9	C5	Li27	48.5	N4	C29	55.3	N1	C29	54.1
N1	C27	70.6	N4	C29	65.4	C3	Li27	67.4	C2	Li27	63.0
			C3	Li27	67.8						

Table S43. Most significant (leading) attractive and repulsive diatomic inter-molecular interactions in transition states **4** formed along four potential reaction pathways (RP). All values in kcal/mol.

Atom A	Atom B	RP-C2	Atom A	Atom B	RP-C3	Atom A	Atom B	RP-C5	Atom A	Atom B	RP-C10
Attractive interactions with $\lvert E_{int}(A,B)\rvert$ > ~10 kcal/mol											
N1	Li27	-201.8	N4	Li27	-199.6	N4	Li27	-189.4	N1	Li27	-195.7
C2	C28	-102.1	C3	C28	-107.1	C5	C28	-115.7	C10	C28	-118.4
N4	Li27	-82.9	N1	Li27	-84.4	N1	Li27	-85.0	N4	Li27	-84.4
C3	C29	-36.7	C2	C29	-33.4	C10	C29	-28.4	C10	C29	-27.4
C2	C29	-30.2	C3	C29	-32.2	C5	C29	-27.0	C5	C29	-27.2
C10	C29	-18.5	C5	C29	-17.7	C3	C29	-21.1	C2	C29	-20.1
C5	C29	-16.0	C10	C29	-16.2	C2	C29	-18.9	C3	C29	-19.3
C3	C28	-14.2	C2	C28	-13.3	C10	C28	-14.0	C5	C28	-13.5
C10	C28	-9.5	C5	C28	-9.8	C3	C28	-13.4	C2	C28	-12.2
Repulsive interactions with $\lvert E_{int}(A,B)\rvert$ > ~10 kcal/mol											
N1	C28	19.4	N1	C28	11.4	N1	C28	12.3	N4	C28	11.5
C5	Li27	27.7	N4	C28	21.2	N4	C28	21.0	N1	C28	20.0
N1	C29	51.9	C10	Li27	30.3	C2	Li27	35.3	C3	Li27	36.1
C3	Li27	52.8	C2	Li27	50.0	C10	Li27	43.9	C5	Li27	42.2
C10	Li27	53.7	N1	C29	50.7	N4	C29	48.6	N1	C29	48.6
N4	C29	53.9	N4	C29	50.9	N1	C29	49.8	N4	C29	49.6
C2	Li27	65.8	C5	Li27	53.5	C5	Li27	55.4	C10	Li27	57.9
			C3	Li27	66.5	C3	Li27	64.9	C2	Li27	63.0

Table S44. Most significant (leading) attractive and repulsive diatomic intra-molecular interactions in intermediates **5** formed along four potential reaction pathways (RP). All values in kcal/mol.

**Atom A**	**Atom B**	**RP-C2**	**Atom A**	**Atom B**	**RP-C3**	**Atom A**	**Atom B**	**RP-C5**	**Atom A**	**Atom B**	**RP-C10**
Attractive interactions with $\|E_{int}(A,B)\|$ > ~10 kcal/mol											
N1	Li27	-220.8	N4	Li27	-223.7	N4	Li27	-215.8	N1	Li27	-214.4
N4	Li27	-77.3	N1	Li27	-77.9	N1	Li27	-77.4	N4	Li27	-76.3
C29	Li27	-13.6	C28	Li27	-13.5	C29	Li27	-13.1	C29	Li27	-16.5
C28	Li27	-12.9	C29	Li27	-13.2	C28	Li27	-12.3	C28	Li27	-14.2
Repulsive interactions with $\|E_{int}(A,B)\|$ > ~10 kcal/mol											
C5	Li27	23.3	C10	Li27	25.3	C2	Li27	29.4	C3	Li27	29.5
C3	Li27	46.0	C2	Li27	44.0	C10	Li27	38.5	C5	Li27	36.5
C2	Li27	47.7	C3	Li27	48.4	C5	Li27	44.8	C10	Li27	46.7
C10	Li27	56.4	C5	Li27	57.5	C3	Li27	67.2	C2	Li27	64.6

Part 4
Net atomic charges

Table S45. Net atomic charges for the reactants **1** and **2**. *Q* is in *e*.

**1**		**2**	
**Atom A**	***Q*(A)**	**Atom A**	***Q*(A)**
N1	-1.1164	Li1	0.9463
C2	0.5418	C2	-0.3217
C3	0.5651	C3	-0.6415
N4	-1.1211	C4	0.0371
C5	0.4269	C5	-0.0256
C6	-0.0137	C6	-0.0297
C7	-0.0156	C7	-0.0343
C8	-0.0154	C8	-0.0297
C9	-0.0150	C9	-0.0255
C10	0.4426	H10	0.0267
H11	0.0542	H11	0.0240
H12	0.0535	H12	0.0234
H13	0.0425	H13	0.0241
H14	0.0426	H14	0.0269
H15	0.0529		
C16	-0.0130		
C17	-0.0144		
C18	-0.0190		
C19	-0.0224		
C20	-0.0179		
C21	-0.0247		
H22	0.0512		
H23	0.0344		
H24	0.0352		
H25	0.0349		
H26	0.0315		

Table S46. Net atomic charges for **H_2O**. *Q* is in *e*.

**H_2O**	
**Atom A**	***Q*(A)**
O1	-1.1385
H2	0.5693
H3	0.5693

Table S47. Net atomic charges for all the stationary points involved in the nucleophilic addition step along RP-C2. *Q* is in *e*.

	***Q*(A)**		
**Atom A**	**3a**	**4a**	**5a**
N1	-1.1725	-1.1849	-1.1814
C2	0.5430	0.4651	0.4036
C3	0.5764	0.6455	0.6338
N4	-1.1166	-1.1589	-1.1803
C5	0.4336	0.3524	0.3175
C6	-0.0090	-0.0256	-0.0322
C7	-0.0113	-0.0390	-0.0565
C8	-0.0090	-0.0306	-0.0391
C9	-0.0153	-0.0483	-0.0640
C10	0.4276	0.4191	0.4363
H11	0.0618	0.0429	0.0332
H12	0.0578	0.0337	0.0203
H13	0.0463	0.0196	0.0044
H14	0.0464	0.0214	0.0077
H15	0.0420	0.0168	-0.0111
C16	-0.0100	-0.0190	-0.0312
C17	-0.0243	-0.0292	-0.0356
C18	-0.0187	-0.0279	-0.0283
C19	-0.0200	-0.0291	-0.0300
C20	-0.0138	-0.0255	-0.0289
C21	-0.0114	-0.0251	-0.0375
H22	0.0378	0.0536	0.0353
H23	0.0343	0.0268	0.0292
H24	0.0361	0.0281	0.0289
H25	0.0367	0.0276	0.0281
H26	0.0450	0.0366	0.0381
Li27	0.9272	0.9360	0.9503
C28	-0.2885	-0.1028	-0.1431
C29	-0.6494	-0.5181	-0.2079
C30	0.0384	0.0492	0.0542
C31	-0.0281	-0.0144	-0.0128
C32	-0.0309	-0.0201	-0.0202
C33	-0.0361	-0.0240	-0.0247
C34	-0.0310	-0.0205	-0.0209
C35	-0.0236	-0.0163	-0.0154
H36	0.0291	0.0423	0.0444
H37	0.0249	0.0351	0.0346
H38	0.0228	0.0345	0.0329
H39	0.0236	0.0342	0.0330
H40	0.0284	0.0386	0.0359

Table S48. Net atomic charges for all the stationary points involved in the nucleophilic addition step along RP-C3. *Q* is in *e*.

	***Q*(A)**		
**Atom A**	**3b**	**4b**	**5b**
N1	-1.1100	-1.1566	-1.1710
C2	0.5471	0.6115	0.6058
C3	0.5421	0.4690	0.4139
N4	-1.1921	-1.1896	-1.1984
C5	0.4189	0.4160	0.4415
C6	-0.0127	-0.0492	-0.0641
C7	-0.0111	-0.0309	-0.0397
C8	-0.0121	-0.0388	-0.0555
C9	-0.0112	-0.0275	-0.0342
C10	0.4472	0.3795	0.3411
H11	0.0975	0.0425	0.0130
H12	0.0451	0.0182	-0.0097
H13	0.0460	0.0217	0.0080
H14	0.0460	0.0196	0.0046
H15	0.0570	0.0326	0.0199
C16	-0.0159	-0.0119	-0.0140
C17	-0.0160	-0.0194	-0.0212
C18	-0.0198	-0.0237	-0.0255
C19	-0.0238	-0.0268	-0.0303
C20	-0.0181	-0.0191	-0.0250
C21	-0.0246	-0.0196	-0.0350
H22	0.0505	0.0537	0.0496
H23	0.0330	0.0263	0.0270
H24	0.0340	0.0286	0.0276
H25	0.0426	0.0270	0.0263
H26	0.0416	0.0240	0.0235
Li27	0.9249	0.9338	0.9518
C28	-0.2816	-0.1180	-0.1559
C29	-0.6516	-0.5158	-0.2095
C30	0.0353	0.0530	0.0547
C31	-0.0234	-0.0152	-0.0158
C32	-0.0278	-0.0209	-0.0215
C33	-0.0340	-0.0242	-0.0252
C34	-0.0282	-0.0191	-0.0208
C35	-0.0256	-0.0088	-0.0131
H36	0.0288	0.0358	0.0355
H37	0.0253	0.0326	0.0324
H38	0.0237	0.0331	0.0322
H39	0.0253	0.0339	0.0339
H40	0.0280	0.0429	0.0433

Table S49. Net atomic charges for all the stationary points involved in the nucleophilic addition step along RP-C5. *Q* is in *e*.

	***Q*(A)**		
**Atom A**	**3c**	**4c**	**5c**
N1	-1.1083	-1.1529	-1.1685
C2	0.5498	0.4370	0.3937
C3	0.5563	0.5009	0.4994
N4	-1.1868	-1.1733	-1.1848
C5	0.4159	0.3979	0.3928
C6	-0.0204	-0.0235	-0.0615
C7	-0.0115	-0.0209	-0.0264
C8	-0.0117	-0.0367	-0.0511
C9	-0.0112	-0.0231	-0.0311
C10	0.4507	0.5346	0.5320
H11	0.0602	0.0300	0.0120
H12	0.0818	0.0238	0.0046
H13	0.0489	0.0232	0.0167
H14	0.0467	0.0238	0.0151
H15	0.0569	0.0356	0.0307
C16	-0.0127	-0.0119	-0.0122
C17	-0.0129	-0.0276	-0.0364
C18	-0.0173	-0.0280	-0.0335
C19	-0.0205	-0.0346	-0.0433
C20	-0.0160	-0.0277	-0.0337
C21	-0.0243	-0.0369	-0.0448
H22	0.0526	0.0447	0.0379
H23	0.0366	0.0237	0.0168
H24	0.0374	0.0234	0.0157
H25	0.0367	0.0229	0.0159
H26	0.0304	0.0138	0.0095
Li27	0.9190	0.9401	0.9555
C28	-0.2954	-0.1135	-0.1450
C29	-0.6456	-0.4937	-0.2108
C30	0.0374	0.0504	0.0539
C31	-0.0262	-0.0149	-0.0140
C32	-0.0301	-0.0207	-0.0211
C33	-0.0349	-0.0258	-0.0253
C34	-0.0301	-0.0214	-0.0216
C35	-0.0263	-0.0167	-0.0153
H36	0.0268	0.0423	0.0424
H37	0.0238	0.0337	0.0335
H38	0.0230	0.0327	0.0320
H39	0.0238	0.0325	0.0325
H40	0.0269	0.0364	0.0377

Table S50. Net atomic charges for all the stationary points involved in the nucleophilic addition step along RP-C10. *Q* is in *e*.

	***Q*(A)**		
**Atom A**	**3d**	**4d**	**5d**
N1	-1.1768	-1.1720	-1.1755
C2	0.5334	0.4860	0.4878
C3	0.5733	0.4421	0.3913
N4	-1.1123	-1.1630	-1.1798
C5	0.4350	0.5160	0.5112
C6	-0.0097	-0.0238	-0.0317
C7	-0.0115	-0.0398	-0.0542
C8	-0.0105	-0.0215	-0.0272
C9	-0.0231	-0.0278	-0.0605
C10	0.4182	0.4086	0.3954
H11	0.0633	0.0224	0.0083
H12	0.0580	0.0346	0.0287
H13	0.0470	0.0220	0.0125
H14	0.0494	0.0221	0.0147
H15	0.0791	0.0206	-0.0027
C16	-0.0191	-0.0250	-0.0266
C17	-0.0262	-0.0267	-0.0424
C18	-0.0142	-0.0260	-0.0281
C19	-0.0175	-0.0284	-0.0299
C20	-0.0142	-0.0240	-0.0258
C21	-0.0215	-0.0298	-0.0298
H22	0.0447	0.0455	0.0295
H23	0.0423	0.0291	0.0279
H24	0.0409	0.0300	0.0283
H25	0.0408	0.0303	0.0297
H26	0.0384	0.0296	0.0315
Li27	0.9183	0.9395	0.9532
C28	-0.2962	-0.1073	-0.1434
C29	-0.6440	-0.4919	-0.2244
C30	0.0371	0.0504	0.0536
C31	-0.0260	-0.0165	-0.0151
C32	-0.0300	-0.0212	-0.0213
C33	-0.0347	-0.0255	-0.0251
C34	-0.0301	-0.0211	-0.0215
C35	-0.0261	-0.0149	-0.0155
H36	0.0269	0.0365	0.0397
H37	0.0239	0.0325	0.0334
H38	0.0231	0.0327	0.0325
H39	0.0238	0.0336	0.0330
H40	0.0268	0.0416	0.0382

Table S51. Net atomic charges for all the stationary points involved in the hydrolysis step along RP-C2. *Q* is in *e*.

	Q(A)		
**Atom A**	**6a**	**7a**	**8a**
N1	-1.1636	-1.1453	-1.1136
C2	0.4041	0.4062	0.4188
C3	0.6331	0.6484	0.6621
N4	-1.1798	-1.1691	-1.1609
C5	0.3177	0.3227	0.3339
C6	-0.0323	-0.0270	-0.0214
C7	-0.0571	-0.0459	-0.0386
C8	-0.0393	-0.0330	-0.0266
C9	-0.0637	-0.0521	-0.0348
C10	0.4426	0.3991	0.3995
H11	0.0343	0.0422	0.0511
H12	0.0199	0.0278	0.0339
H13	0.0039	0.0124	0.0178
H14	0.0076	0.0158	0.0219
H15	-0.0094	0.0218	0.0323
C16	-0.0277	-0.0220	-0.0202
C17	-0.0332	-0.0412	-0.0299
C18	-0.0282	-0.0283	-0.0303
C19	-0.0299	-0.0282	-0.0312
C20	-0.0281	-0.0255	-0.0273
C21	-0.0339	-0.0262	-0.0285
H22	0.0405	0.0308	0.0446
H23	0.0282	0.0310	0.0315
H24	0.0281	0.0311	0.0311
H25	0.0270	0.0310	0.0315
H26	0.0337	0.0404	0.0431
Li27	0.9324	0.9496	0.9471
C28	-0.1473	-0.1415	-0.1364
C29	-0.2232	-0.1989	-0.1937
C30	0.0553	0.0556	0.0562
C31	-0.0097	-0.0111	-0.0103
C32	-0.0177	-0.0193	-0.0183
C33	-0.0221	-0.0235	-0.0227
C34	-0.0188	-0.0200	-0.0188
C35	-0.0146	-0.0135	-0.0118
H36	0.0479	0.0448	0.0455
H37	0.0373	0.0353	0.0362
H38	0.0355	0.0337	0.0347
H39	0.0353	0.0341	0.0349
H40	0.0366	0.0386	0.0390
O41	-1.2050	-1.2891	-1.3796
H42	0.5919	0.5620	0.4965
H43	0.5924	0.5462	0.5117

Table S52. Net atomic charges for all the stationary points involved in the hydrolysis step along RP-C3. Q is in e.

	***Q*(A)**		
**Atom A**	**6b**	**7b**	**8b**
N1	-1.1716	-1.1625	-1.1566
C2	0.6092	0.6235	0.6375
C3	0.4108	0.4163	0.4238
N4	-1.1789	-1.1696	-1.1236
C5	0.4468	0.3973	0.3946
C6	-0.0635	-0.0504	-0.0372
C7	-0.0394	-0.0328	-0.0282
C8	-0.0563	-0.0439	-0.0387
C9	-0.0341	-0.0285	-0.0239
C10	0.3418	0.3456	0.3546
H11	0.0193	0.0279	0.0479
H12	-0.0086	0.0233	0.0345
H13	0.0077	0.0171	0.0208
H14	0.0042	0.0134	0.0174
H15	0.0196	0.0278	0.0326
C16	-0.0141	-0.0151	-0.0131
C17	-0.0206	-0.0189	-0.0163
C18	-0.0253	-0.0228	-0.0214
C19	-0.0302	-0.0270	-0.0255
C20	-0.0246	-0.0218	-0.0201
C21	-0.0352	-0.0324	-0.0299
H22	0.0504	0.0521	0.0544
H23	0.0271	0.0302	0.0312
H24	0.0276	0.0314	0.0323
H25	0.0260	0.0304	0.0315
H26	0.0222	0.0278	0.0296
Li27	0.9328	0.9477	0.9486
C28	-0.1562	-0.1526	-0.1492
C29	-0.2267	-0.1991	-0.1946
C30	0.0535	0.0558	0.0564
C31	-0.0143	-0.0135	-0.0125
C32	-0.0194	-0.0203	-0.0196
C33	-0.0227	-0.0240	-0.0232
C34	-0.0186	-0.0195	-0.0190
C35	-0.0111	-0.0117	-0.0109
H36	0.0385	0.0375	0.0394
H37	0.0351	0.0336	0.0343
H38	0.0350	0.0333	0.0339
H39	0.0366	0.0348	0.0353
H40	0.0462	0.0441	0.0445
O41	-1.2105	-1.2955	-1.3812
H42	0.5962	0.5488	0.5144
H43	0.5951	0.5632	0.4957

Table S53. Net atomic charges for all the stationary points involved in the hydrolysis step along RP-C2. *Q* is in *e*.

	***Q*(A)**		
**Atom A**	**6a**	**7e**	**8e**
N1	-1.1636	-1.1725	-1.1495
C2	0.4041	0.5561	0.5939
C3	0.6331	0.6512	0.5974
N4	-1.1798	-1.1453	-1.1144
C5	0.3177	0.3684	0.4336
C6	-0.0323	-0.0204	-0.0080
C7	-0.0571	-0.0320	-0.0114
C8	-0.0393	-0.0248	-0.0083
C9	-0.0637	-0.0365	-0.0072
C10	0.4426	0.4130	0.4444
H11	0.0343	0.0525	0.0699
H12	0.0199	0.0399	0.0587
H13	0.0039	0.0259	0.0478
H14	0.0076	0.0269	0.0486
H15	-0.0094	0.0158	0.0492
C16	-0.0277	-0.2200	-0.0282
C17	-0.0332	-0.0465	-0.0259
C18	-0.0282	-0.0456	-0.0238
C19	-0.0299	-0.0434	-0.0268
C20	-0.0281	-0.0469	-0.0279
C21	-0.0339	-0.0491	-0.0201
H22	0.0405	-0.0019	0.0247
H23	0.0282	0.0109	0.0266
H24	0.0281	0.0141	0.0277
H25	0.0270	0.0102	0.0251
H26	0.0337	0.0015	0.0294
Li27	0.9324	0.9344	0.9156
C28	-0.1473	-0.1370	-0.1193
C29	-0.2232	-0.1592	-0.1665
C30	0.0553	0.0579	0.0561
C31	-0.0097	-0.0115	-0.0101
C32	-0.0177	-0.0178	-0.0160
C33	-0.0221	-0.0229	-0.0208
C34	-0.0188	-0.0185	-0.0188
C35	-0.0146	-0.0118	-0.0284
H36	0.0479	0.0418	0.0438
H37	0.0373	0.0360	0.0378
H38	0.0355	0.0348	0.0378
H39	0.0353	0.0347	0.0376
H40	0.0366	0.0363	0.1137
O41	-1.2050	-1.2387	-1.4254
H42	0.5919	0.5653	0.0314
H43	0.5924	0.5761	0.5069

Part 5
Inter-fragment interaction energies

Interaction energies computed for adducts 3

Table S54. Interaction energies between molecule **1** and specified major fragments of molecule **2**, computed for the indicated reaction pathways on adducts **3** formation between **1** and **2**. All values in kcal/mol.

		**Reaction pathway**			
**Fragment**		**C2**	**C3**	**C5**	**C10**
**1**	**2**	-56.6	-64.0	-45.0	-47.4
	ℛ	-19.7	-25.4	-5.5	-5.5
	𝒫𝒽2	-17.4	-13.0	-0.5	-0.5
	ℒ	-39.2	-51.0	-44.5	-46.9
	𝒜	-2.3	-12.3	-5.0	-5.0
	Li	-36.9	-38.6	-39.4	-41.9
	C28	-1.1	-3.6	-3.6	-4.2
	C29	-1.2	-8.7	-1.4	-0.8

Table S55. Interaction energies between molecule **2** and specified major fragments of molecule **1**, computed for the indicated reaction pathways on adducts **3** formation between **1** and **2**. All values in kcal/mol.

		**Reaction pathway**			
**Fragment**		**C2**	**C3**	**C5**	**C10**
**2**	𝒬	-43.5	-50.5	-46.6	-47.3
	𝒫	-46.5	-54.5	-39.9	-40.2
	ℬ𝓃	23.0	27.7	17.1	17.0
	𝒫𝒽1	-13.2	-13.6	1.6	-0.1
	𝒩	-83.0	-92.0	-103.4	-100.9
	𝒞	36.7	45.4	61.1	59.5
	𝒢1	-43.3	0.9	1.5	-43.1
	𝒢2	-3.0	-47.5	-43.9	1.8
	ℱ1	-59.8	11.3	21.9	-51.6
	ℱ2	7.6	-67.7	-48.4	20.9
	ℱ3	11.4	-57.8	-65.4	8.8
	ℱ4	-54.4	13.5	8.7	-62.6
	N1	-74.1	-10.5	-15.4	-85.2
	N4	-8.9	-81.5	-88.1	-15.6
	C2	15.0	5.2	10.0	24.9
	C3	1.6	16.6	27.2	10.5
	C5	4.2	17.4	16.9	6.9
	C10	15.7	6.2	7.0	17.2

Table S56. Interaction energies, between the molecular fragment $\mathcal{R}^{a}$ of **2** and the indicated significant fragments of **1**, computed for the indicated reaction pathways on adducts **3** formation between **1** and **2**. All values in kcal/mol.

		Reaction pathway			
Fragment		C2	C3	C5	C10
$\mathcal{R}$	$\mathcal{Q}$	-0.5	-5.7	-1.3	-0.3
	$\mathcal{P}$	9.1	3.1	22.1	23.3
	$\mathcal{Bn}$	-70.7	-63.9	-80.8	-80.0
	$\mathcal{Ph}$1	-19.2	-19.7	-4.2	-5.2
	$\mathcal{N}$	162.7	161.5	151.3	149.9
	$\mathcal{C}$	-149.1	-139.3	-125.2	-123.4
	$\mathcal{G}$1	17.3	1.1	1.2	25.9
	$\mathcal{G}$2	-3.6	21.1	24.9	0.6
	$\mathcal{F}$1	12.7	-25.6	-14.8	31.5
	$\mathcal{F}$2	-30.8	0.2	26.7	-17.6
	$\mathcal{F}$3	0.5	48.6	41.5	-4.0
	$\mathcal{F}$4	41.0	2.8	-4.8	41.5
	N1	100.9	58.1	52.8	97.3
	N4	61.8	103.4	98.5	52.6
	C2	-48.8	-32.9	-27.7	-38.1
	C3	-39.1	-51.3	-40.2	-29.0
	C5	-26.3	-31.0	-33.4	-23.0
	C10	-34.8	-24.1	-23.9	-33.3

[a]) $\mathcal{R}$ = {C28,C29,C30,C31,C32,C33,C34,C35,H36,H37,H38.H39.H40}

Table S57. Interaction energies, between the molecular fragment 𝒫𝒽2[a] of **2** and the indicated significant fragments of **1**, computed for the indicated reaction pathways on adducts **3** formation between **1** and **2**. All values in kcal/mol.

		**Reaction pathway**			
**Fragment**		**C2**	**C3**	**C5**	**C10**
**𝒫𝒽2**	𝒬	-7.6	-0.8	-0.6	-0.7
	𝒫	-8.0	-1.1	-1.0	-1.0
	ℬ𝓃	3.1	2.6	2.4	2.5
	𝒫𝒽1	-9.8	-12.3	0.1	0.2
	𝒩	-10.3	-7.0	-5.6	-5.8
	𝒞	2.7	5.6	4.5	4.6
	𝒢1	-2.6	0.0	0.0	-1.2
	𝒢2	-5.0	-1.5	-1.1	0.0
	ℱ1	-6.1	1.2	0.7	-1.5
	ℱ2	-5.2	-1.5	-1.3	0.8
	ℱ3	-2.0	-2.9	-2.0	0.3
	ℱ4	-2.9	0.4	0.3	-2.0
	N1	-5.6	-1.9	-1.7	-4.0
	N4	-4.7	-5.1	-3.9	-1.8
	C2	1.3	1.0	0.9	1.5
	C3	-1.4	2.2	1.5	1.0
	C5	1.1	1.4	1.3	0.8
	C10	1.6	0.9	0.8	1.3

[a]) 𝒫𝒽2 = {C30,C31,C32,C33,C34,C35,H36,H37,H38.H39.H40}

Table S58. Interaction energies, between the molecular fragment $\mathcal{L}$[a] of **2** and the indicated significant fragments of **1**, computed for the indicated reaction pathways on adducts **3** formation between **1** and **2**. All values in kcal/mol.

		Reaction pathway			
**Fragment**		**C2**	**C3**	**C5**	**C10**
$\mathcal{L}$	$\mathcal{Q}$	-35.9	-49.7	-46.0	-46.6
	$\mathcal{P}$	-38.5	-53.4	-38.9	-39.1
	$\mathcal{Bn}$	19.9	25.1	14.7	14.5
	$\mathcal{Ph}$1	-3.3	-1.3	1.5	-0.3
	$\mathcal{N}$	-72.7	-85.0	-97.8	-95.1
	$\mathcal{C}$	34.0	39.9	56.6	54.9
	$\mathcal{G}$1	-40.7	0.9	1.5	-42.0
	$\mathcal{G}$2	2.0	-46.0	-42.8	1.7
	$\mathcal{F}$1	-53.7	10	21.1	-50.1
	$\mathcal{F}$2	12.8	-66.2	-47.1	20.1
	$\mathcal{F}$3	13.5	-55.0	-63.4	8.5
	$\mathcal{F}$4	-51.5	13.1	8.4	-60.6
	N1	-68.6	-8.6	-13.7	-81.3
	N4	-4.1	-76.4	-84.2	-13.8
	C2	13.7	4.1	9.0	23.4
	C3	3.0	14.3	25.7	9.5
	C5	3.2	16.0	15.6	6.1
	C10	14.1	5.4	6.2	15.9

[a])$\mathcal{L}$ = {Li28,C28,C29}

Table S59. Interaction energies, between the molecular fragment $\mathcal{A}$[a] of **2** and the indicated significant fragments of **1**, computed for the indicated reaction pathways on adducts **3** formation between **1** and **2**. All values in kcal/mol.

		Reaction pathway			
**Fragment**		**C2**	**C3**	**C5**	**C10**
$\mathcal{A}$	$\mathcal{Q}$	7.0	-4.9	-0.7	0.3
	$\mathcal{P}$	17.1	4.3	23.1	24.3
	$\mathcal{Bn}$	-73.8	-66.5	-83.2	-82.5
	$\mathcal{Ph}$1	-9.3	-7.5	-4.3	-5.3
	$\mathcal{N}$	173.0	168.5	156.9	155.6
	$\mathcal{C}$	-151.8	-144.8	-129.7	-128.0
	$\mathcal{G}$1	19.9	1.1	1.2	27.0
	$\mathcal{G}$2	1.4	22.6	26.0	0.6
	$\mathcal{F}$1	18.9	-26.9	-15.5	33.0
	$\mathcal{F}$2	-25.6	1.7	28.0	-18.4
	$\mathcal{F}$3	2.6	51.5	43.5	-4.4
	$\mathcal{F}$4	43.9	2.4	-5.1	43.5
	N1	106.5	60.0	54.5	101.2
	N4	66.5	108.5	102.4	54.4
	C2	-50.2	-33.9	-28.6	-39.6
	C3	-37.8	-53.5	-41.7	-30.0
	C5	-27.4	-32.4	-34.7	-23.8
	C10	-36.5	-25.0	-24.7	-34.7

[a]) $\mathcal{A}$ = {C28,C29}

Table S60. Interaction energies, between Li27 of **2** and the indicated significant fragments of **1**, computed for the indicated reaction pathways on adducts **3** formation between **1** and **2**. All values in kcal/mol.

		Reaction pathway			
**Fragment**		**C2**	**C3**	**C5**	**C10**
**Li27**	$\mathcal{Q}$	-42.9	-44.8	-45.5	-47.0
	$\mathcal{P}$	-55.6	-57.6	-62.0	-63.5
	$\mathcal{Bn}$	93.7	91.6	97.9	97.0
	$\mathcal{Ph}$1	6.0	6.2	5.8	5.1
	$\mathcal{N}$	-245.7	-253.4	-254.7	-250.7
	$\mathcal{C}$	185.7	184.7	186.3	182.9
	$\mathcal{G}$1	-60.6	-0.2	0.4	-69.0
	$\mathcal{G}$2	0.6	-68.6	-68.8	1.1
	$\mathcal{F}$1	-72.5	36.9	36.6	-83.1
	$\mathcal{F}$2	38.4	-67.8	-75.1	38.5
	$\mathcal{F}$3	10.9	-106.4	-106.9	12.8
	$\mathcal{F}$4	-95.3	10.8	13.5	-104.1
	N1	-175.0	-68.6	-68.2	-182.5
	N4	-70.7	-184.9	-186.6	-68.2
	C2	63.9	38.1	37.7	63.0
	C3	40.8	67.8	67.4	39.5
	C5	30.5	48.5	50.3	29.9
	C10	50.6	30.3	30.9	50.6

Table S61. Interaction energies, between C28 of **2** and the indicated significant fragments of **1**, computed for the indicated reaction pathways on adducts **3** formation between **1** and **2**. All values in kcal/mol.

		Reaction pathway			
**Fragment**		**C2**	**C3**	**C5**	**C10**
**C28**	$\mathcal{Q}$	1.9	-2.2	-1.8	-1.9
	$\mathcal{P}$	5.2	1.3	11.0	11.9
	$\mathcal{Bn}$	-24.0	-24.7	-38.1	-39.0
	$\mathcal{Ph}1$	-2.9	-1.5	-1.8	-2.4
	$\mathcal{N}$	52.8	62.1	68.7	68.9
	$\mathcal{C}$	-46.7	-52.6	-55.8	-55.7
	$\mathcal{G}1$	6.7	0.0	0.0	13.4
	$\mathcal{G}2$	-0.5	9.5	12.8	-0.2
	$\mathcal{F}1$	9.5	-12.2	-8.9	17.3
	$\mathcal{F}2$	-10.0	3.5	14.7	-10.0
	$\mathcal{F}3$	-3.8	22.0	21.2	-3.7
	$\mathcal{F}4$	15.8	-2.3	-3.8	21.2
	N1	35.9	19.0	21.6	47.1
	N4	17.0	43.1	47.1	21.8
	C2	-16.3	-10.6	-11.7	-18.0
	C3	-9.7	-20.8	-18.9	-12.4
	C5	-7.7	-12.8	-15.4	-9.6
	C10	-12.9	-8.4	-9.8	-15.7

Table S62. Interaction energies, between C29 of **2** and the indicated significant fragments of **1**, computed for the indicated reaction pathways on adducts **3** formation between **1** and **2**. All values in kcal/mol.

		Reaction pathway			
**Fragment**		**C2**	**C3**	**C5**	**C10**
**C29**	$\mathcal{Q}$	5.2	-2.7	1.1	2.2
	$\mathcal{P}$	11.9	3.0	12.1	12.5
	$\mathcal{Bn}$	-49.9	-41.8	-45.1	-43.5
	$\mathcal{Ph}$1	-6.4	-6.0	-2.6	-3.0
	$\mathcal{N}$	120.2	106.4	88.2	86.7
	$\mathcal{C}$	-105.1	-92.2	-73.9	-72.3
	$\mathcal{G}$1	13.1	1.1	1.1	13.6
	$\mathcal{G}$2	1.9	13.1	13.2	0.8
	$\mathcal{F}$1	9.4	-14.7	-6.6	15.7
	$\mathcal{F}$2	-15.5	-1.8	13.3	-8.5
	$\mathcal{F}$3	6.4	29.5	22.3	-0.7
	$\mathcal{F}$4	28.0	4.7	-1.3	22.3
	N1	70.6	40.9	32.9	54.1
	N4	49.6	65.4	55.3	32.6
	C2	-33.9	-23.3	-16.9	-21.5
	C3	-28.1	-32.7	-22.8	-17.6
	C5	-19.6	-19.6	-19.3	-14.2
	C10	-23.6	-16.5	-14.9	-19.0

Interaction energies computed for transition states 4 of the nucleophilic addition step

Table S63. Interaction energies between molecule **1** and specified major fragments of molecule **2**, computed for the indicated reaction pathways on transition states **4** formation between **1** and **2**. All values in kcal/mol.

		Reaction pathway			
Fragment		C2	C3	C5	C10
**1**	**2**	-206.8	-209.7	-207.5	-216.6
	$\mathcal{R}$	-130.7	-137.0	-144.1	-144.6
	$\mathcal{Ph}$2	-5.2	-11.2	-6.3	-6.2
	$\mathcal{L}$	-201.6	-198.4	-201.2	-210.4
	$\mathcal{A}$	-125.5	-125.8	-137.7	-13.4
	Li	-76.1	-72.6	-63.5	-72.0
	C28	-118.1	-114.7	-133	-135.5
	C29	-7.3	-11.1	-4.7	-3.0

Table S64. Interaction energies, between Li27 of **2** and the indicated significant fragments of **1**, computed for the indicated reaction pathways on transition states **4** formation between **1** and **2**. All values in kcal/mol.

		Reaction pathway			
Fragment		C2	C3	C5	C10
**Li27**	$\mathcal{Q}$	-80.6	-77.5	-64.1	-73.0
	$\mathcal{P}$	-80.8	-77.5	-70.8	-78.5
	$\mathcal{Bn}$	81.6	83.7	106.0	105.5
	$\mathcal{Ph}$1	4.5	4.9	0.7	1.0
	$\mathcal{N}$	-284.7	-284.0	-274.4	-280.1
	$\mathcal{C}$	200.0	200.3	199.4	199.1
	$\mathcal{G}$1	-82.2	-4.1	-5.8	-74.8
	$\mathcal{G}$2	-2.4	-79.6	-69.2	-6.1
	$\mathcal{F}$1	-86.0	31.9	14.9	-100.4
	$\mathcal{F}$2	39.6	-76.9	-85.1	17.2
	$\mathcal{F}$3	-1.9	-120.4	-90.4	15.3
	$\mathcal{F}$4	-124.6	-1.3	14.0	-96.5
	N1	-201.8	-84.4	-85.0	-195.7
	N4	-82.9	-199.6	-189.4	-84.4
	C2	65.8	50.0	35.3	63.0
	C3	52.8	66.5	64.9	36.1
	C5	27.7	53.5	55.4	42.2
	C10	53.7	30.3	43.9	57.9

Table S65. Interaction energies, between the molecular fragment $\mathcal{A}$[a] of **2** and the indicated significant fragments of **1**, computed for the indicated reaction pathways on transition states **4** formation between **1** and **2**. All values in kcal/mol.

		Reaction pathway			
Fragment		C2	C3	C5	C10
$\mathcal{A}$	$\mathcal{Q}$	-104.6	-113.0	-136.7	-136.7
	$\mathcal{P}$	-102.7	-111.2	-117.7	-118.6
	$\mathcal{Bn}$	-50.8	-51.3	-204.0	-204.6
	$\mathcal{Ph}$1	-20.9	-12.8	-1.0	-1.7
	$\mathcal{N}$	133.5	134.2	131.6	129.7
	$\mathcal{C}$	-232.2	-235.7	-247.0	-246.5
	$\mathcal{G}$1	-89.1	-6.7	-7.8	-109.5
	$\mathcal{G}$2	-9.7	-94.8	-107.7	-7.3
	$\mathcal{F}$1	-117.5	-126.0	0.2	9.5
	$\mathcal{F}$2	-125.1	-123.8	5.2	-0.7
	$\mathcal{F}$3	13.5	23.9	-123.6	-126.3
	$\mathcal{F}$4	23.7	12.9	-123.9	-125.9
	N1	71.3	62.1	62.1	68.6
	N4	62.2	72.1	69.5	61.1
	C2	-132.3	-46.7	-27.5	-32.3
	C3	-51.0	-139.4	-34.5	-27.7
	C5	-20.9	-27.5	-142.7	-40.7
	C10	-28.0	-22.0	-42.3	-145.8

[a]) $\mathcal{A}$ = {C28,C29}

Table S66. Interaction energies, between the molecular fragment $\mathcal{R}$[a] of **2** and the indicated significant fragments of **1**, computed for the indicated reaction pathways on transition states **4** formation between **1** and **2**. All values in kcal/mol.

		Reaction pathway			
Fragment		C2	C3	C5	C10
$\mathcal{R}$	$\mathcal{Q}$	-109.7	-118.2	-142.8	-143.2
	$\mathcal{P}$	-107.5	-116.2	-122.9	-124.0
	$\mathcal{Bn}$	-43.1	-43.0	-194.0	-194.7
	$\mathcal{Ph}1$	-21.0	-18.8	-1.2	-1.5
	$\mathcal{N}$	106.6	106.1	105.9	103.9
	$\mathcal{C}$	-210.8	-213.2	-226.8	-226.4
	$\mathcal{G}1$	-92.4	-8.9	-10.6	-112.3
	$\mathcal{G}2$	-11.8	-98.2	-110.4	-10.2
	$\mathcal{F}1$	-117.7	-127.9	-3.8	5.9
	$\mathcal{F}2$	-124.4	-123.1	2.2	-4.4
	$\mathcal{F}3$	7.9	18.1	-126.0	-129.1
	$\mathcal{F}4$	17.8	7.1	-126.6	-128.3
	N1	57.9	47.9	49.0	55.9
	N4	48.7	58.2	56.9	48.0
	C2	-126.6	-38.9	-23.3	-27.5
	C3	-43.3	-133.3	-29.5	-23.3
	C5	-17.3	-23.1	-137.8	-34.9
	C10	-23.7	-18.0	-36.2	-140.7

[a]) $\mathcal{R}$ = {C28,C29,C30,C31,C32,C33,C34,C35,H36,H37,H38.H39.H40}

Table S67. Interaction energies, between the molecular fragment 𝒫𝒽2[a] of **2** and the indicated significant fragments of **1**, computed for the indicated reaction pathways on transition states **4** formation between **1** and **2**. All values in kcal/mol.

		Reaction pathway			
**Fragment**		**C2**	**C3**	**C5**	**C10**
**𝒫𝒽2**	𝒬	-5.0	-5.2	-6.1	-6.4
	𝒫	-4.8	-5.0	-5.2	-5.5
	ℬ𝓃	7.8	8.3	10.0	9.9
	𝒫𝒽1	-0.2	-6.0	-0.2	0.2
	𝒩	-26.8	-28.1	-25.7	-25.
	𝒞	21.4	22.5	20.2	20.0
	𝒢1	-3.3	-2.2	-2.8	-2.8
	𝒢2	-2.1	-3.4	-2.7	-3.0
	ℱ1	-0.2	-1.8	-4.0	-3.7
	ℱ2	0.6	0.7	-3.0	-3.7
	ℱ3	-5.7	-5.3	-2.3	-2.8
	ℱ4	-5.8	-5.9	-2.7	-2.4
	N1	-13.4	-14.9	-13.1	-12.6
	N4	-13.4	-13.9	-12.6	-13.1
	C2	5.7	7.9	4.2	4.8
	C3	7.7	6.1	5.0	4.4
	C5	3.6	4.4	4.9	5.8
	C10	4.4	4.1	6.1	5.1

[a]) 𝒫𝒽2 = {C30,C31,C32,C33,C34,C35,H36,H37,H38.H39.H40}

Table S68. Interaction energies, between C28 of **2** and the indicated significant fragments of **1**, computed for the indicated reaction pathways on transition states **4** formation between **1** and **2**. All values in kcal/mol.

		Reaction pathway			
**Fragment**		**C2**	**C3**	**C5**	**C10**
**C28**	$\mathcal{Q}$	-105.3	-110.4	-132.2	-134.8
	$\mathcal{P}$	-104.0	-109.3	-119.3	-121.6
	$\mathcal{Bn}$	-15.7	-16.8	-142.6	-145.0
	$\mathcal{Ph}$1	-12.8	-4.2	-0.8	-0.7
	$\mathcal{N}$	27.6	32.6	33.3	31.5
	$\mathcal{C}$	-130.7	-136.1	-151.7	-152.5
	$\mathcal{G}$1	-92.2	-7.7	-10.3	-110.6
	$\mathcal{G}$2	-10.9	-95.8	-108.2	-10.4
	$\mathcal{F}$1	-102.2	-110.6	-9.9	-0.3
	$\mathcal{F}$2	-109.0	-105.1	-2.0	-9.8
	$\mathcal{F}$3	-6.3	5.8	-116.0	-121.8
	$\mathcal{F}$4	5.1	-4.3	-118.7	-119.3
	N1	19.4	11.4	12.3	20.0
	N4	8.2	21.2	21.0	11.5
	C2	-102.1	-13.3	-8.7	-12.2
	C3	-14.2	-107.1	-13.4	-8.4
	C5	-4.9	-9.8	-115.7	-13.5
	C10	-9.5	-5.8	-14.0	-118.4

Table S69. Interaction energies, between C29 of **2** and the indicated significant fragments of **1**, computed for the indicated reaction pathways on transition states **4** formation between **1** and **2**. All values in kcal/mol.

		**Reaction pathway**			
**Fragment**		**C2**	**C3**	**C5**	**C10**
**C29**	$\mathcal{Q}$	0.7	-2.6	-4.5	-2.0
	$\mathcal{P}$	1.3	-1.9	1.6	3.1
	$\mathcal{Bn}$	-35.2	-34.6	-61.4	-59.6
	$\mathcal{Ph}$1	-8.0	-8.6	-0.2	-1.0
	$\mathcal{N}$	105.8	101.6	98.4	98.2
	$\mathcal{C}$	-101.5	-99.6	-95.3	-93.9
	$\mathcal{G}$1	3.2	1.1	2.5	1.1
	$\mathcal{G}$2	1.2	1.0	0.5	3.2
	$\mathcal{F}$1	-15.3	-15.4	10.1	9.9
	$\mathcal{F}$2	-16.1	-18.7	7.2	9.1
	$\mathcal{F}$3	19.9	18.1	-2.3	-4.5
	$\mathcal{F}$4	18.5	17.2	-2.7	-6.6
	N1	51.9	50.7	49.8	48.6
	N4	53.9	50.9	48.6	49.6
	C2	-30.2	-33.4	-18.9	-20.1
	C3	-36.7	-32.2	-21.1	-19.3
	C5	-16.0	-17.7	-27.0	-27.2
	C10	-18.5	-16.2	-28.4	-27.4

PART 6

Energy profiles along reaction coordinates

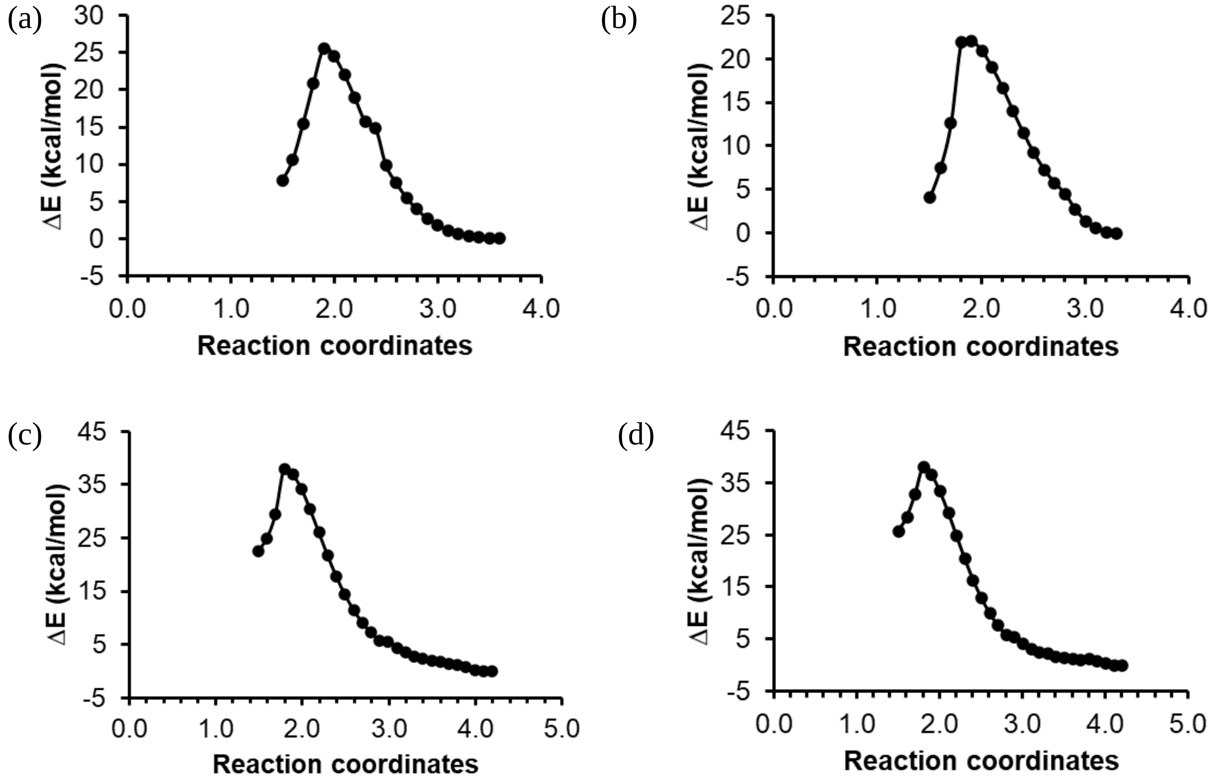

Figure S1. Computed reaction energy profiles along the C28---Cn reaction coordinates for (a) RP-C2, (b) RP-C3, (c) RP-C5 and (d) RP-C10.

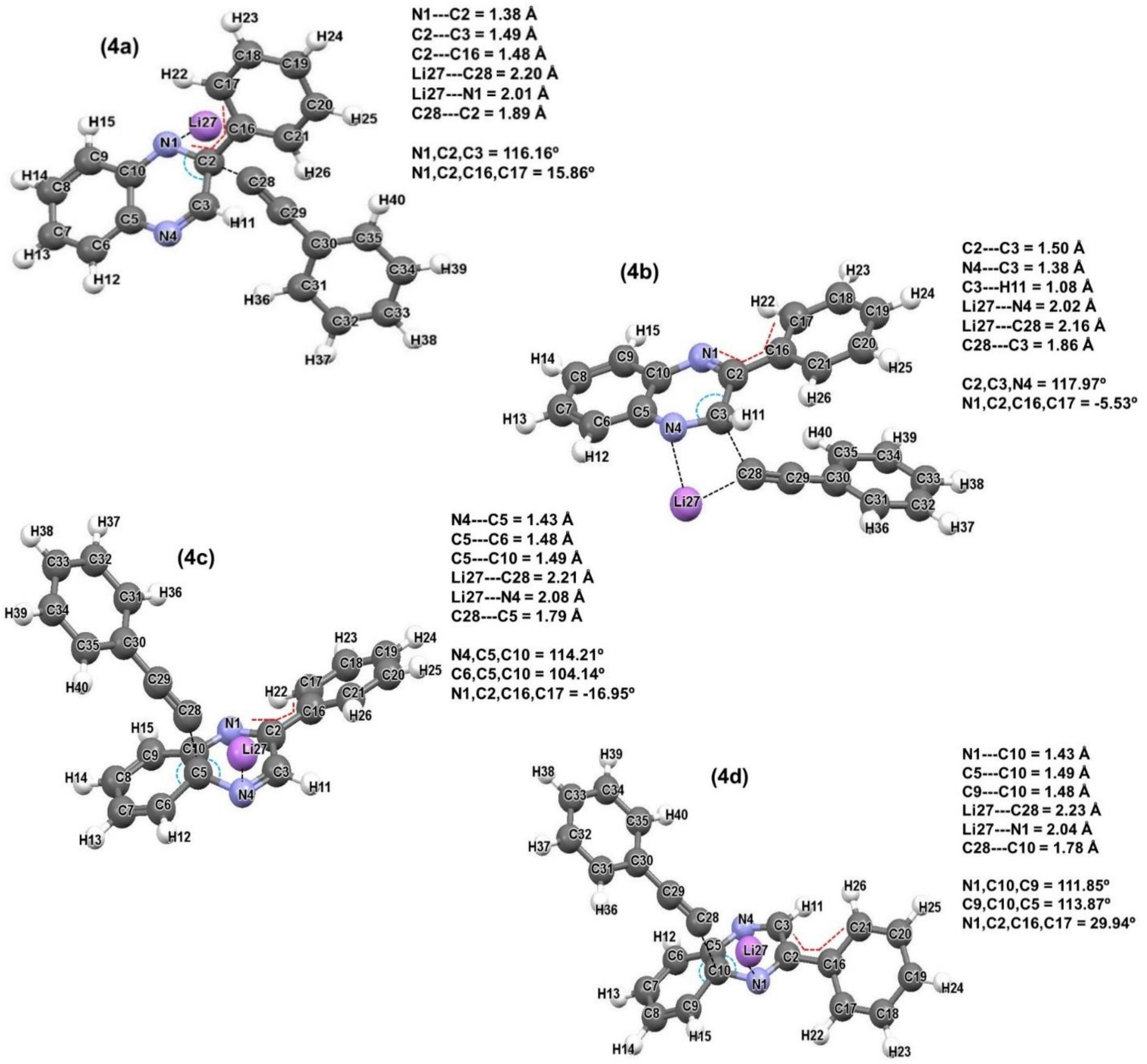

Figure S2. Energy-optimized electronic structures of the TSs (**4a-d**) along all RPs at the nucleophilic addition stage.

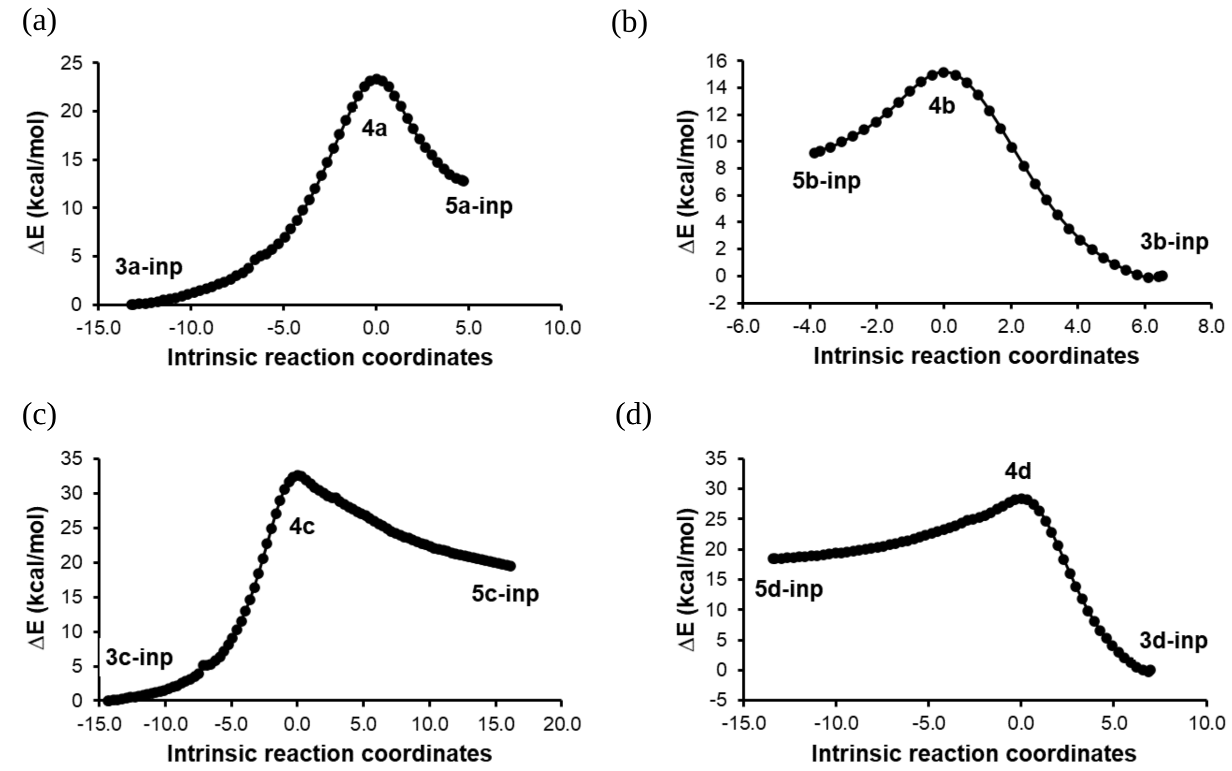

Figure S3. Computed IRC paths for the nucleophilic addition step along (a) RP-C2, (b) RP-C3, (c) RP-C5 and (d) RP-C10, confirming that **4a**, **4b**, **4c** and **4d** connect the desired minima of the respective pathways.

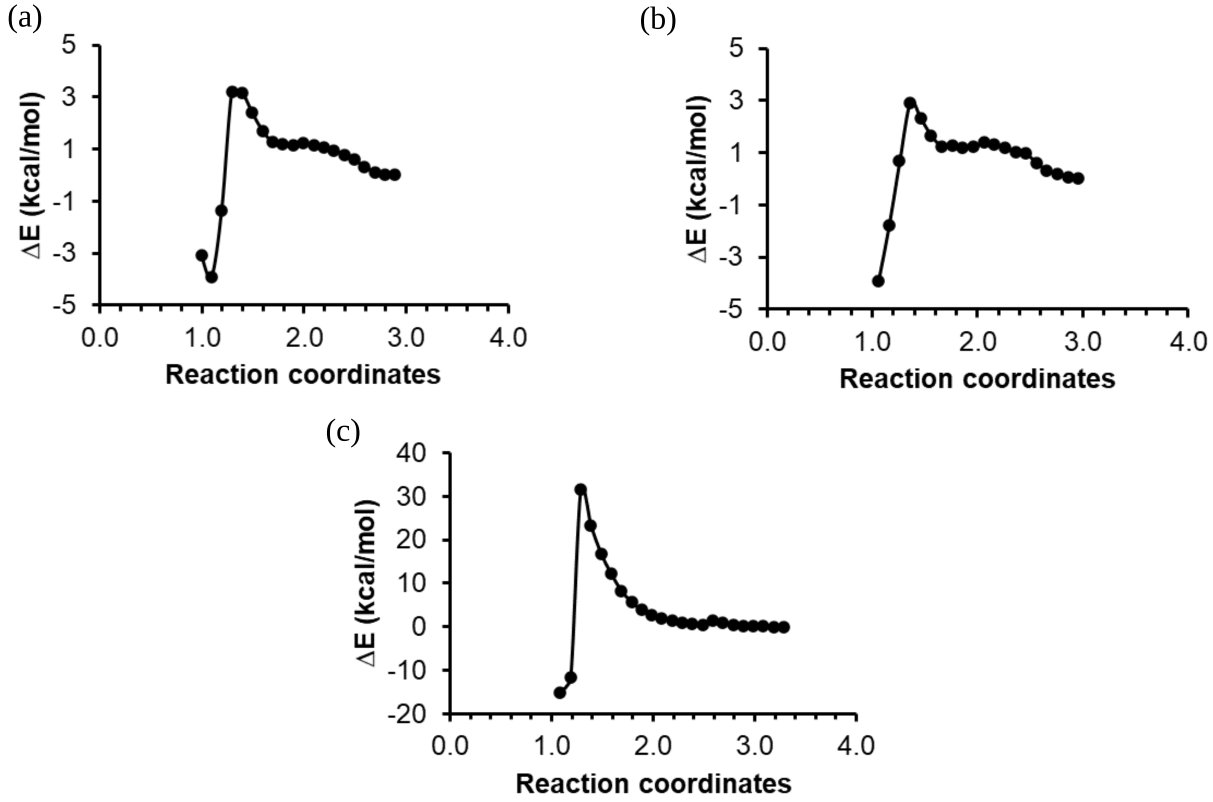

Figure S4. Computed reaction energy profiles slong (a) RP-C2, when H42 is transferred to N1 (b) RP-C3, when H43 is transferred to N4 and (c) RP-C2, when H42 is transferred to C16.

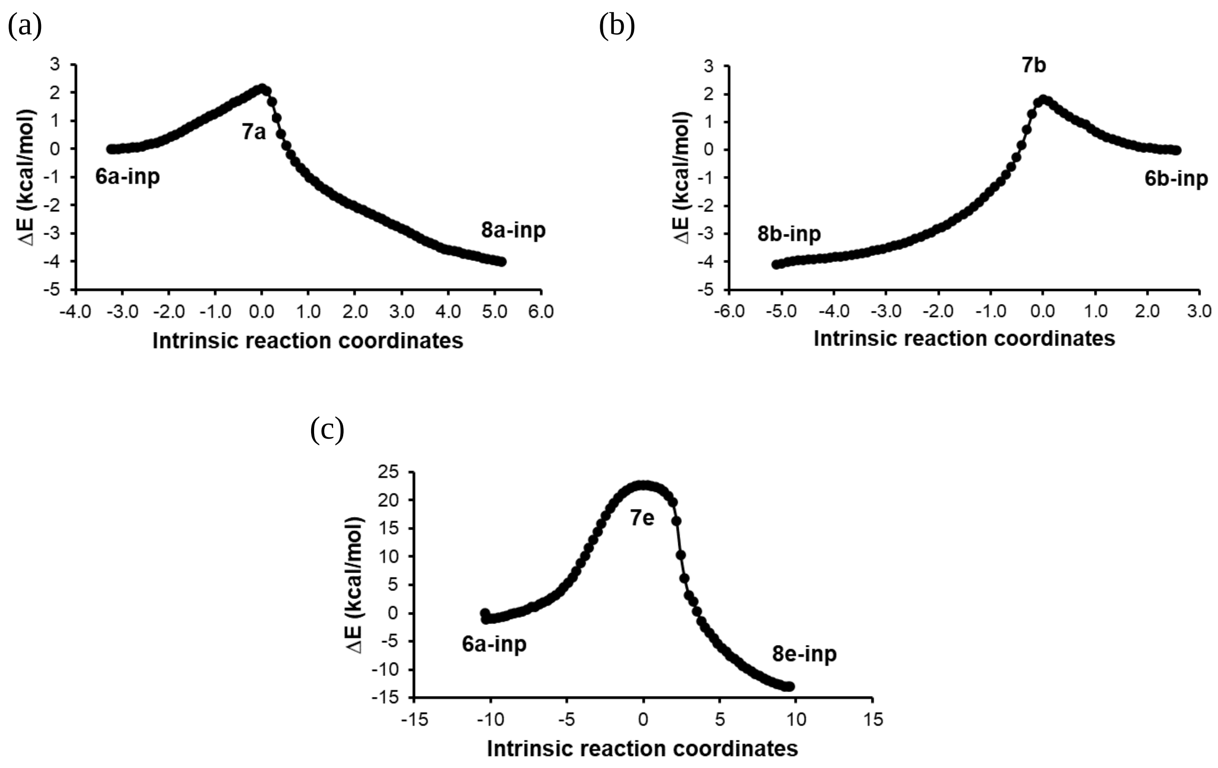

Figure S5. Computed IRC paths for the hydrolysis step along (a) RP-C2, when H42 is transferred to N1 (b) RP-C3, when H43 is transferred to N4 and (c) RP-C2, when H42 is transferred to C16.
